# Understanding changes in the locations of drinking occasions in Great Britain: An age‐period‐cohort analysis of repeat cross‐sectional market research data, 2001–2019

**DOI:** 10.1111/dar.13562

**Published:** 2022-10-12

**Authors:** Iain Hardie, Alessandro Sasso, John Holmes, Petra S. Meier

**Affiliations:** ^1^ MRC/CSO Social and Public Health Sciences Unit University of Glasgow Glasgow UK; ^2^ Sheffield Alcohol Research Group, School of Health and Related Research University of Sheffield Sheffield UK

**Keywords:** age‐period‐cohort analysis, alcohol, drinking locations, drinking practices, Great Britain

## Abstract

**Introduction:**

The 21st century has seen wide‐ranging changes in drinking locations in Great Britain, with on‐trade alcohol sales decreasing and off‐trade sales increasing. To better understand the underlying time‐trends in consumer behaviour, we examine age‐period‐cohort (APC) effects related to changes in the share of individuals' drinking occasions taking place in: (i) on‐trade versus off‐trade locations; and (ii) specific on‐trade or off‐trade locations, that is traditional/community pubs, modern pubs/bars/café bars, nightclubs/late‐night venues, restaurants/pub restaurants, social/working men's clubs, golf/other sports clubs/venues, at home (social setting) and at home (non‐social setting).

**Methods:**

Repeat cross‐sectional 1‐week drinking diary data, collected 2001–2019. APC analysis via negative binomial regression models for each gender (*N* = 162,296 men, 138,452 women).

**Results:**

A smaller/declining proportion of occasions took place in on‐trade compared to off‐trade locations. Recent cohorts tended to have a larger share of on‐trade occasions than previous cohorts, driven by their larger share of occasions in modern pubs/bars/café bars and nightclubs/late‐night venues. Meanwhile, occasions in social/working men's clubs, golf/other sports clubs/venues and traditional/community pubs tended to be popular among older men, but have declined. Finally, the growth of off‐trade drinking appears to be driven by a growth of off‐trade drinking in non‐social settings, in particular by older people/cohorts.

**Discussion and Conclusion:**

Our findings highlight the declining prominence of certain on‐trade locations, and increasing prominence of home drinking in non‐social settings, within British drinking practices. While rising non‐social home drinking is concerning, it is positive from a public health perspective that it does not appear to be shared by recent cohorts.

## 1 INTRODUCTION

There have been wide‐ranging changes to alcohol consumption in Great Britain in the 21st century. Total per‐capita consumption rose to a historic peak in 2004, before subsequently declining steeply [1]. This decline has not been equal across the population. Britain, like many high‐income countries, has seen a notable decline in drinking among young people [2, 3, 4, 5], but not among middle‐aged/older people [6, 7, 8]. Recent decades have also seen a closing of the gender gap in alcohol consumption/harm [9, 10].

Recent decades have also seen changes in how people distribute their drinking across different locations. Sales data suggests that on‐trade sales (i.e., from pubs, bars, clubs and restaurants) have decreased, while off‐trade sales (i.e., from supermarkets and off‐licences) have increased [11]. Similarly, analysis of the spatial availability of outlets selling alcohol suggests that the density of off‐trade outlets increased, and on‐trade outlets decreased from 2003 to 2013 [12]. This is backed up by data showing that the total number of UK pubs/bars fell from 52,500 in 2001 to 39,130 in 2019 [13].

The drivers of this on‐trade to off‐trade shift are currently not fully understood. One potential explanation relates to affordability. It has been noted that cheaper competition from supermarkets encourages people to increase the share of their consumption occurring at home [14]. Meanwhile, rising production costs and taxation have increased on‐trade prices in particular [15]. The introduction of smoking bans in public places may also have reduced on‐trade drinking, as some evidence suggests that they have decreased on‐trade alcohol consumption/expenditure among smokers [16, 17]. Other explanations relate to changing social norms. This includes reduced acceptability of drink‐driving, changing workplace cultures (including longer/less structured working days and reduced lunchtime or post‐work drinking) and an increasing number of health‐conscious young people abstaining from alcohol altogether [18, 19, 20]. More broadly, changes in on‐trade drinking practices, particularly in urban areas, may be attributed to the decline of traditional industries (associated with strong workplace drinking cultures) and the rise of the service sector, with traditional pubs and working men's clubs often being replaced with modern bars [21]. Importantly, these kinds of shifts in drinking locations can have implications for public health, as rates of alcohol‐related harm vary between locations [22].

A popular method for examining alcohol‐related trends is age‐period‐cohort (APC) analysis. The goal of APC analysis is to support understanding of changes in a given outcome of interest over time, with the rationale being that such changes can be attributed to three distinct processes: (i) ‘age effects’, that is, variations associated with chronological age; (ii) period effects, that is, variations over time periods or calendar years influencing all age‐groups simultaneously; and (iii) cohort effects, that is, changes across groups of individuals born in the same year or years [23, 24].

UK‐based APC studies on alcohol tend to suggest strong age and cohort effects. For example, one study suggests that rising alcohol consumption from 1994 to 2004 was linked to older lower‐consuming cohorts being replaced in the population by more recent higher‐consuming cohorts, while more recent declines in consumption are linked to increased abstinence among recent cohorts [5]. Other UK research suggests that countervailing alcohol consumption and alcohol‐related harm trends in the UK may be due to differing APC trends between lighter and heavier drinkers [8]. Internationally, APC studies on alcohol consumption and harm [25, 26, 27, 28] also indicate strong age and cohort effects. This suggests that heavier or lighter drinking birth cohorts moving through periods of life course associated with heavier or lighter drinking practices will have a large impact on population‐level alcohol consumption [8, 26].

While the above studies focus on alcohol consumption/harm, there are currently no known APC studies focussing on drinking locations, as few long‐running data series provide information on this. We address this by making use of repeat cross‐section market research data collected by market research company Kantar to conduct an APC analysis on changes in drinking occasion locations in Great Britain (2001–2019). Men and women are analysed separately as previous research highlights gender differences in: (i) location/beverage preferences (e.g., men tend to dominate on‐trade beer drinking while women dominate off‐trade wine drinking) [29]; and (ii) APC trends in alcohol consumption [5, 8]. The specific research questions addressed are as follows:

How does the share of men's and women's drinking occasions taking place in on‐trade and off‐trade locations differ by age, period and cohort?

How does the share of men's and women's drinking occasions taking place in specific on‐trade and off‐trade locations differ by age, period and cohort?

## 2 METHODS

### 2.1 Study design

APC analysis of repeat cross‐sectional market research data from Kantar Alcovision (2001–2019). Our analysis plan was pre‐registered using the Open Science Framework (available from: https://osf.io/tvrw2/).

### 2.2 Data and participants

Since 2009, Alcovision has drawn monthly quota samples defined by age, sex, social class and geographic region from Kantar's online managed access panel (with recruitment made via recruitment drives). Previously, from 2001 to 2008, Alcovision used on‐street sampling with the same quota‐based approach (with recruitment made via approaching all walkers‐by in different locations). To deal with potential representativeness issues due to the use of quota sampling, we compute sampling weights using a ‘raking’ technique that adjusts the marginal distribution of the target variables to align with UK census data (for full details see Appendix S1, Supporting Information). Alcovision is made up of a short introductory questionnaire and a detailed 7‐day retrospective drinking diary in which respondents report their alcohol consumption and drinking occasion characteristics (including location).

Alcovision's annual sample size in our analysis period was approximately 12,500 under on‐street sampling (2001–2008) and approximately 20,000 under online sampling (2009–2019). Our final analytical sample across the entire 2001–2019 analysis period was 300,748 adult drinkers in England, Scotland or Wales. Alcohol abstainers (*N* = 43,615) were excluded from the analysis. Birth cohorts from before 1930 (*N* = 3175) and after 1999 (*N* = 2862) were also excluded from the main analysis due to their small sample sizes and only being observed at very young or old ages.

### 2.3 Measures

#### 2.3.1 APC measures

Age is categorised into five unequal intervals: 18–24, 25–34, 35–49, 50–64 and 65+ years. This deviates from the pre‐registered analysis plan which measured those aged 65–74 and 75+ years separately. This is because of low sample size for the 75+ group. Period is categorised into calendar years. Birth cohort is categorised into seven decades: 1930s, 1940s, 1950s, 1960s, 1970s, 1980s and 1990s. Categorising APC measures into categorical groupings like this disrupts the exact linear dependence that leads to the APC ‘identification problem’ (i.e., the fact that cohort + age = period) [30].

#### 2.3.2 Outcome measures

We use Alcovision's drinking diary component to create outcomes measuring the individual‐level share (%) of drinking occasions occurring in various locations. First, the share of individuals' occasions taking place in: (i) on‐trade locations; and (ii) off‐trade locations is measured. Second, the share of individuals' occasions taking place in the following specific on‐trade and off‐trade locations is measured: (i) traditional/community pub; (ii) modern pub/bar/café bar; (iii) nightclub/late‐night venue; (iv) restaurant/pub restaurant; (v) social/working men's club; (vi) golf/other sports club/venue; (vii) at home (social setting); and (viii) at home (non‐social setting). These are defined as the share (%) of each respondent's occasions which include drinking in each location. The ‘at home’ measures include occasions in own homes, holiday homes or someone else's home. They are separated into social or non‐social settings by using additional information on the reported purpose of the occasion. ‘Social setting’ includes occasions reported as ‘sociable night in/catch‐up/planned sociable occasion’, ‘a big night in/party/special occasion’, ‘a barbeque/picnic’ or ‘friends/family unplanned occasion/having friends round’. ‘Non‐social setting’ includes occasions reported as ‘regular evening/lunchtime/everyday drink’, ‘rounding off the evening’ or ‘just having a drink/drink after work/quiet night in’. Occasions taking place in student pubs/union bars, hotels, cinemas/theatres, bowling/bingo/leisure venues, outdoors, at festivals/events or in unknown/unlisted venues were all excluded from the specific locations analysis due to low prevalence (<2% of overall occasions).

### 2.4 Statistical analysis

First, APC trends are analysed via descriptive analysis, whereby the weighted mean of each outcome is plotted by gender and APC variables. Second, APC effects are formally modelled for each outcome using negative binomial regression models. Negative binomial regression is a commonly used method within APC studies in alcohol literature [5, 25, 27], and was appropriate for use here as our outcome measures were over‐dispersed [31, 32].

In each model, APC measures are included as explanatory variables, with the 35–49 age‐group, 2009 period and 1960s cohort being reference categories. To account for the aforementioned switch from on‐street to online data collection between 2008 and 2009 a binary control variable is also included in each model (coded 0 = on‐street data collection, 1 = online data collection). To remove perfect collinearity between the period measure and the binary control for the change in data collection, 2008 is omitted as a category from the period measure (for full details see Appendix S1).

Two sensitivity analyses are carried out. First, in the main analysis, we assumed the data can be treated as a single time‐series despite the change in data collection method between 2008 and 2009. To test this assumption, the main analysis is repeated with 2001–2008 and 2009–2019 modelled separately. Second, a key challenge of all APC studies relates to the aforementioned ‘identification problem’. Our analysis follows other similar studies [5, 8, 33] in disrupting linear dependence by categorising APC measures into differential time grouping. To ensure our APC estimates are robust and consistent, our second sensitivity analysis repeats the main analysis with differently specified APC variables (i.e., using different APC groupings), as recommended by APC literature [30].

## 3 RESULTS

### 3.1 Descriptive statistics and analysis

**FIGURE 1:**
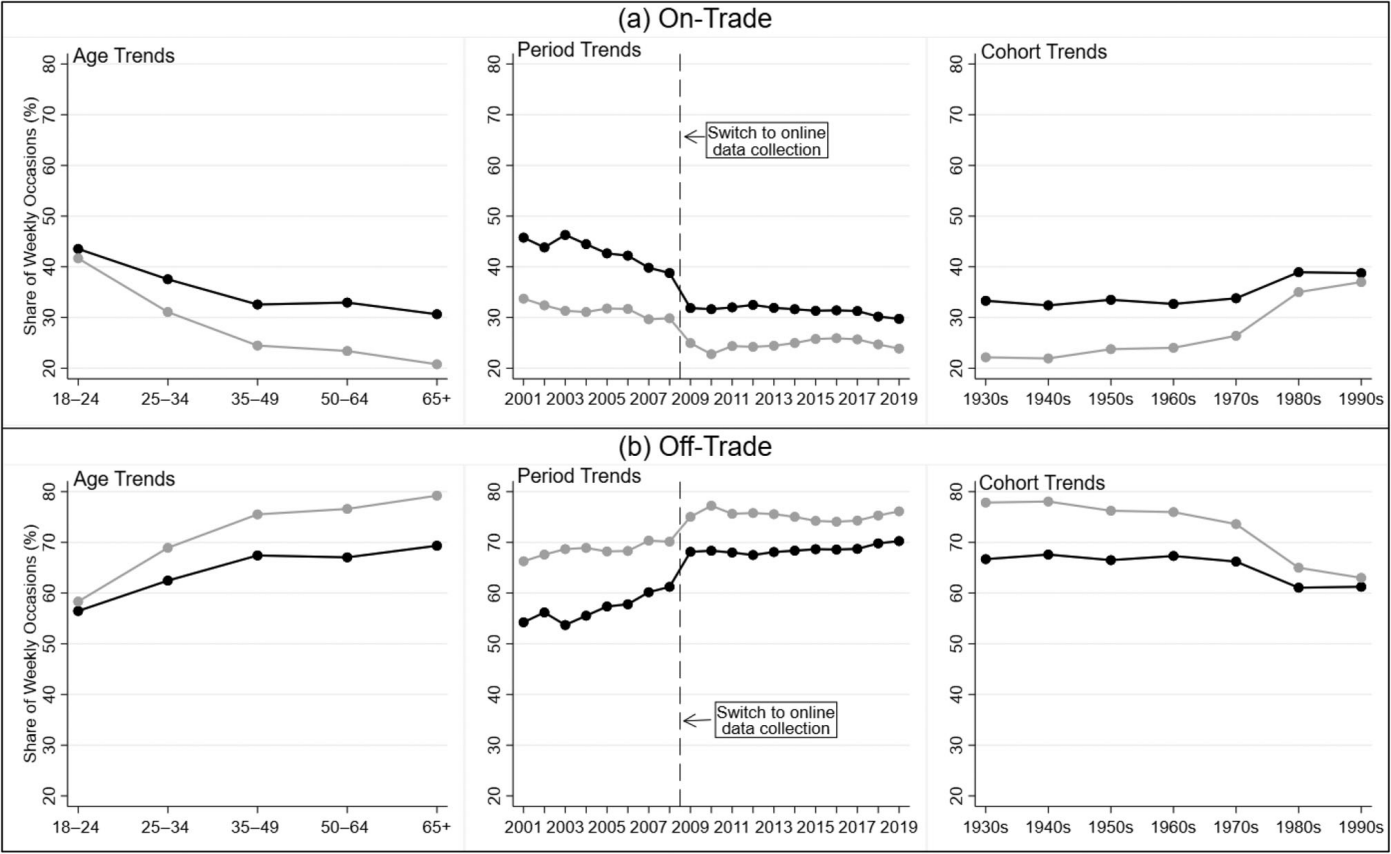
Descriptive analysis: age, period and cohort trends in the mean percentage share of men and women's individual drinking occasions per week taking place in on‐trade versus off‐trade locations, 2001–2019. Blue: men; orange: women.

**FIGURE 2:**
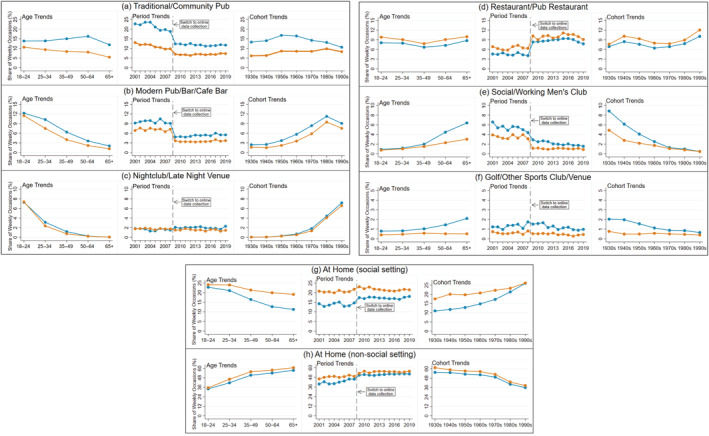
Descriptive analysis: age, period and cohort trends in the mean percentage share of men and women's individual drinking occasions per week taking place in specific on‐trade and off‐trade locations, 2001–2019. Blue: men; orange: women. *Notes*: Locations have varying y‐axis ranges to best depict results.

Descriptive statistics showing the final analytical sample by APC variables and gender are provided in Table 1. APC trends by gender are plotted in Figures 1 and 2. Period trends here include reference lines signifying Alcovision's switch to online data collection between 2008 and 2009, as this is not controlled for in the descriptive analysis (but is in later negative binomial regression models).

**TABLE 1:** Description of analytic study sample by age, period and cohort (unweighted)

	Men (total *n* = 162,296)	Women (total *n* = 138,452)
Age, years		
18–24	29,895	37,259
25–34	34,279	33,796
35–49	48,105	37,107
50–64	31,524	22,161
65+	18,493	8129
Period		
2001	6633	5275
2002	6209	5067
2003	6032	4727
2004	6078	4859
2005	6102	5133
2006	6185	5264
2007	5794	5048
2008	5464	4468
2009	10,586	9638
2010	10,409	9460
2011	10,719	9451
2012	10,774	9306
2013	10,880	9245
2014	10,958	9410
2015	10,626	9212
2016	10,684	8970
2017	9081	7900
2018	9808	8159
2019	9274	7860
Cohort		
1930s	6091	3258
1940s	17,548	9889
1950s	22,965	15,869
1960s	28,977	22,022
1970s	34,501	26,524
1980s	36,606	35,716
1990s	15,608	25,174

#### 3.1.1 APC trends: On‐trade versus off‐trade

The share of on‐trade occasions tended to be higher among men and young people, and decreased during the analysis period, particularly during the 2000s (see Figure 1).

#### 3.1.2 APC trends: Specific on‐trade and off‐trade locations

By gender, the share of occasions in traditional/community pubs, modern pubs/bars/café bars, social/working men's clubs and golf/other sports clubs/venues was higher among men, while the share of occasions in restaurants/pub restaurants and at home was higher among women (see Figure 2). By age, the share of occasions in modern pubs/bars/café bars, nightclubs/late‐night venues, at home (social setting) and in traditional/community pubs (for women only) tended to be higher among younger age‐groups, while the share of occasions in social/working men's clubs, at home (non‐social setting), in golf/other sports clubs/venues and in traditional/community pubs tended to be higher among older men. By period, the share of occasions in traditional/community pubs, social/working men's clubs and golf/other sports clubs/venues (from 2008 only) declined during the analysis period, while there was a notable increase in the share of occasions at home (particularly in non‐social settings). Finally, by cohort, the share of occasions in modern pubs/bars/café bars, nightclubs/late‐night venues and at home (social setting) were higher among recent cohorts, whereas the share of occasions in social/working men's clubs, golf/other sports clubs/venues and at home (non‐social setting) were higher among earlier cohorts.

### 3.2 APC modelling

**FIGURE 3:**
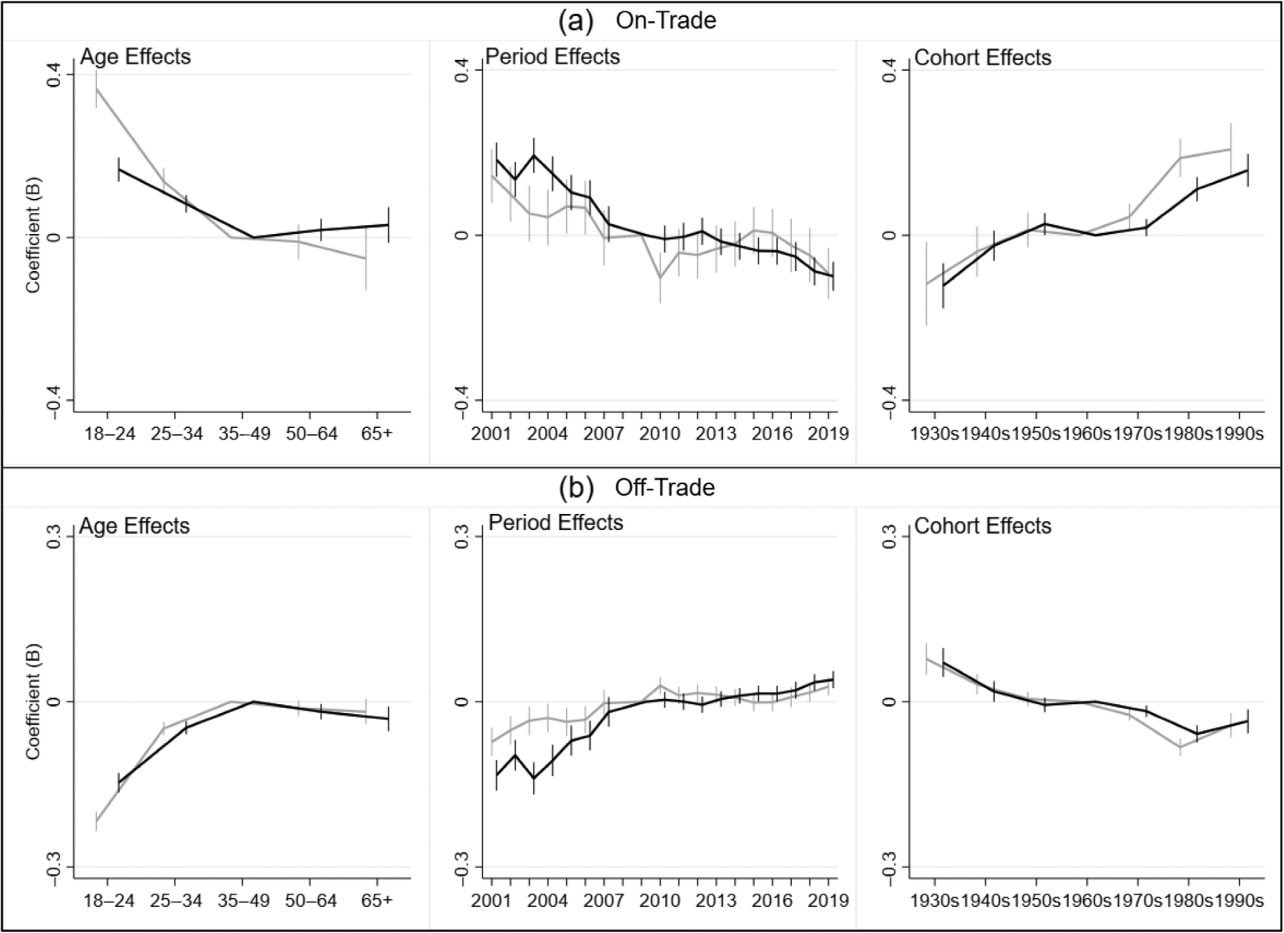
Age‐period‐cohort modelling: age, period and cohort effects on the percentage share of men and women's individual drinking occasions per week taking place in on‐trade versus off‐trade locations. Blue: men; orange: women. *Notes*: Different locations have different y‐axis ranges to best depict results. Reference categories: 35–49, 2009 and 1960s. Vertical bars represent 95% confidence intervals at each data point, and can be interpreted as statistically significant compared to the reference category where they do not cross zero. The year 2008 was omitted from the negative binomial regression models to remove perfect collinearity between the period variable and the binary control variable for the change in data collection between 2008 and 2009.

**FIGURE 4:**
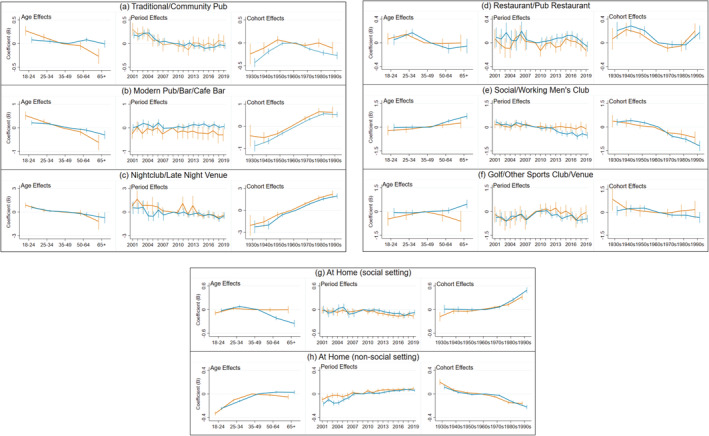
Age‐period‐cohort modelling: age, period and cohort effects on the percentage share of men and women's individual drinking occasions per week taking place in specific on‐trade and off‐trade locations. Blue: men; orange: women. *Notes*: Different locations have different y‐axis ranges to best depict results. Reference categories: 35–49, 2009 and 1960s. Vertical bars represent 95% confidence intervals at each data point, and can be interpreted as statistically significant compared to the reference category where they do not cross zero. The year 2008 was omitted from the negative binomial regression models to remove perfect collinearity between the period variable and the binary control variable for the change in data collection between 2008 and 2009.

The negative binomial regression modelling results are shown in Figure 3 (modelled version of the descriptive analysis in Figure 1) and Figure 4 (modelled version of the descriptive analysis in Figure 2). Detailed model results are also provided in table form in Appendix S2 (Supporting Information).

#### 3.2.1 APC effects: On‐trade versus off‐trade

##### Age effects

Overall, the modelling suggests that the share of on‐trade occasions decreased, and off‐trade occasions increased, throughout the first half of the life course, before remaining stable during the second half of the life course (see Figure 3).

##### Period effects

Period effects suggest the share of occasions in the on‐trade decreased during the analysis period (particularly from 2001 to 2006).

##### Cohort effects

Cohort effects suggest that, in general, the share of occasions in the on‐trade was higher among more recent cohorts.

#### 3.2.2 APC effects: Specific on‐trade and off‐trade locations

##### Age effects

The share of occasions in traditional/community pubs tended to decrease with age for women (see Figure 4). For men, it decreased throughout the first half of the life course only, before then rising to a peak at ages 50–64. Meanwhile, the share of occasions in both modern pubs/bars/café bars and nightclubs/late‐night venues tended to consistently decrease throughout the life course, while the share of occasions in restaurants/pub restaurants peaked at ages 25–34 before decreasing later in the life course. The share of occasions in social/working men's clubs and golf/other sports clubs/venues did not significantly change throughout the life course for women, but for men (who dominate these occasion types) were higher among those aged 65+. Finally, the share of occasions at home (social setting) was fairly stable throughout the life course for women, but for men peaked at ages 25–34 before decreasing later in the life course, while at home (non‐social setting) tended to increase during the first half of the life course before subsequently stabilising from ages 35–49 onwards.

##### Period effects

The share of occasions in traditional/community pubs was in decline in the first half of the analysis period, and subsequently continued to decline among men but increased among women from 2013, although none of these period effects in the latter part of the analysis period were statistically significant compared to the reference category (2009). The share of occasions in modern pubs/bars/café bars was fairly stable throughout the analysis period, while there was a small decrease in the share of occasions in nightclubs/late‐night venues. There was no clear pattern in period effects relating to restaurants/pub restaurants, which tended to fluctuate up and down every few years. Meanwhile, there was a consistent decline in the share of occasions in social/working men's clubs, and this was statistically significant among men (but not women—this likely reflects the fact that it made up such a small proportion of women's occasions in the first place). Following a rise in the 2000s, there was also a statistically significant and consistent decline in the share of men's occasions in golf/other sports clubs/venues in the 2010s. Finally, the share of occasions at home (social setting) was fairly stable during 2001 to 2011 but subsequently declined slightly, while the share of at‐home (non‐social setting) occasions increased consistently.

##### Cohort effects

Cohort effects suggest the share of occasions in traditional/community pubs was highest among the 1950s–1960s cohorts, whereas the share of occasions in modern pubs/bars/café bars and in nightclubs/late‐night venues was highest among recent cohorts. Meanwhile, the share of occasions in restaurants/pub restaurants was highest among pre‐1960s cohorts and the 1990s cohort, while the share of occasions in social/working men's clubs and in golf/other sports clubs/venues was highest among older cohorts. Finally, the share of occasions at home (social setting) was highest among recent cohorts, and at home (non‐social setting) highest among older cohorts.

### 3.3 Summary table of APC findings

An overall summary of the findings from all of the above analyses is provided in Table 2.

**TABLE 2:** Summary of age‐period‐cohort (APC) analysis findings related to the share of occasions in each location

Location	APC variable	APC effects: changes in the share of drinking occasions in each location by age, period and cohort
On‐trade	Age	Decreases with age in the first half of life course then remains stable in the second half of life course.
	Period	Decrease over time, particularly in the first half of analysis period and particularly for men, with gender gap narrowing.
	Cohort	Higher among more recent cohorts.
Off‐trade	Age	Increases with age in the first half of life course then remains stable in the second half of life course.
	Period	Increase over time, particularly during the first half of analysis period. Gender gap narrowing over time.
	Cohort	Higher among older birth cohorts.
Traditional/community pub	Age	Decreases with age in women. In men, small decrease with age in the first half of life course before rising to a peak aged 50–64.
	Period	Decrease over time, particularly during the first half of the analysis period and particularly among men.
	Cohort	Highest among 1950s and 1960s birth cohorts.
Modern pub/bar/café bar	Age	Decreases with age.
	Period	Stable over time.
	Cohort	Higher among more recent cohorts.
Nightclub/late‐night venue	Age	Decreases with age.
	Period	Slight decrease over time.
	Cohort	Higher among more recent cohorts.
Restaurant/pub restaurant	Age	Fairly stable across age groups, with a slight peak among 25–34‐year‐olds.
	Period	Fairly stable over time.
	Cohort	Higher among the pre 1960s cohorts and 1990s cohort.
Social/working men's club	Age	Higher in older age groups, particularly men.
	Period	Consistent decrease over time.
	Cohort	Higher in earlier birth cohorts.
Golf/other sports club/venue	Age	Higher among older men, no significant age effects for women.
	Period	Slight increase in the first half of analysis period. Decreasing since 2009.
	Cohort	Most popular among men from earlier birth cohorts.
At home (social setting)	Age	For men, higher in younger people. For women, also slightly higher for younger people but age effects not significant.
	Period	Fairly stable over time, with slight decline towards the end of analysis period.
	Cohort	Higher among more recent cohorts
At home (non‐social setting)	Age	Higher among older people.
	Period	Increase over time.
	Cohort	Higher among older cohorts.

### 3.4 Sensitivity analysis

The results of the two sensitivity analyses are provided in Appendices 3 and 4. Their findings are consistent with the findings of the main analysis.

## 4 DISCUSSION AND CONCLUSION

The study is the first known APC analysis of drinking locations. The results provide novel insights into changes in the distribution of British drinking occasions across the on‐trade and off‐trade, and across specific on‐trade and off‐trade locations.

First, our findings highlight that a smaller, and declining, the proportion of drinking occurs in the on‐trade than the off‐trade. This is in line with existing analysis of sales data [11] and with analysis mapping the spatial availability of on‐trade and off‐trade outlets selling alcohol [12]. Beyond this, our findings suggest that, while young people are drinking less than previous cohorts [2, 3, 4, 5], when they do drink they have a greater proportion of their occasions in the on‐trade. This appears to be driven in particular by their larger share of occasions in modern pubs/bars/café bars and nightclubs/late‐night venues. This provides some insight into what the decline of youth drinking looks like at a practice level, with more drinking in modern venues and less in traditional pubs. This may be explained by cultural changes, whereby the culture of male‐dominated heavier drinking (which was well suited to traditional pubs) among previous cohorts is being replaced by a culture of lighter drinking in mixed‐sex groups containing a mixture of drinkers and non‐drinkers (which is better suited to modern bars/café bars).

While the on‐trade generally remains a young person's place, certain on‐trade settings (notably social/working men's clubs, golf/other sports clubs/venues and traditional/community pubs) have tended to be more popular among older men. The share of occasions in these locations has declined throughout (at least parts of) the 21st century. This suggests that the shift away from on‐trade drinking in recent decades is likely to be linked to reduced drinking in these specific locations, while the share of occasions in locations like modern pubs/bars/café bars which are popular among young people have remained fairly constant.

Importantly, our findings suggest that the on‐trade locations that in the past have had the greatest gender differences (particularly the male‐dominated occasions in traditional/community pubs and social/working men's clubs) have seen the largest period effects. As a result of these changes, gender differences in location preferences have narrowed over time, and this is likely to have contributed to the recent closing of the gender gap in alcohol consumption and harm [9, 10]. One potential explanation for this is that traditional male drinking practices have broken down as men have become more involved in family life and the pub has become less of a ‘refuge’ from that. In light of this, male‐dominated venues have struggled to maintain their relevance and economic viability, meaning that men have become less able to drink in them even if they wanted to.

Our findings also suggest that the recent growth in the share of off‐trade drinking reflects a particular growth of drinking in non‐social settings. This is driven by older cohorts, while more recent cohorts appear much less likely to engage in non‐social drinking at home. This has implications for public health because, while drinking in a social setting is often linked to enhancing positive emotions, drinking in a non‐social setting (particularly solitary drinking) is associated with coping with negative emotions [34]. Moreover, home drinking tends to be more habitual and routinised than on‐trade drinking [35, 36], and qualitative research with heavy drinkers highlights that being in the home produces opportunities for, and removes barriers to, intoxication [37]. However, our findings suggest that recent cohorts are less likely to engage in non‐social home drinking, and this is positive from a public health perspective given that non‐social solitary drinking in young adulthood is associated with alcohol problems in later adulthood [38]. In the long‐term, the health benefits of recent cohorts comparative lack of non‐social home drinking occasions do, to some extent, depend on whether this preference continues in the future as they age. This remains to be seen, although evidence suggests that recent cohorts do appear to be unique in their healthier approach to alcohol [2, 4].

The key strength of this analysis is that it is based on a very large sample, with drinking occasions data from over 300,000 individuals across 19 years. Importantly, the use of Alcovision data allows for aggregated trends to be disentangled into their component parts, which reveals nuances that would not otherwise be revealed in other data sources (e.g., sales data). However, there are some limitations. First, our data use quota sampling from an online panel, which can lead to biases [39, 40]. Second, despite our comparatively long time‐series of 19 years, our data is still limited in that each birth cohort is only captured at a limited range of ages, which reduces our ability to fully separate age effects from cohort effects. Finally, our analysis is limited by Alcovision's change in data collection methods between 2008 and 2009, and (like all APC analyses) the ‘identification problem’. However, our sensitivity analyses suggest that these latter two points had minimal effects on our results.

With regard to policy, policymakers may seize the opportunity of large‐scale and long‐term changes in the on‐trade sector to shape policy in ways that benefit public health. This may include a gradual reduction in overall alcohol availability but also targeted reductions in venues that promote heavier drinking and increases in venues that promote moderate consumption and minimise harm [41, 42]. In terms of future research implications, this study has focused on the distribution of drinking occasions across locations. Future research may also consider consumption levels. We used data from Great Britain only. Similar research from other countries would be useful for cross‐country comparisons. Finally, our finding of increased off‐trade drinking, and non‐social home drinking in particular, highlights the increasing importance of the home as a drinking location. Despite recent attempts to redress the balance [36, 37], home drinking currently remains under‐researched compared to on‐trade drinking [43]. Going forward, researchers should place a larger focus on home drinking.

## AUTHOR CONTIBUTIONS

**Iain Hardie:** Conceptualisation; formal analysis; methodology; writing ‐ original draft. **Alessandro Sasso:** Data curation; writing ‐ review and editing. **Petra S. Meier:** Conceptualisation; funding acquisition; project administration; writing ‐ review and editing. **John Holmes:** Conceptualisation; funding acquisition; project administration; writing ‐ review and editing.

## CONFLICT OF INTEREST

The authors declare no conflict of interest.

## ETHICS STATEMENT

As this was an analysis of secondary data no university ethical approval was required. Use of the data was allowed under the terms of the contract and non‐disclosure agreement between Kantar and the Universities of Sheffield and Glasgow.

## Supporting information


**Appendix S1.** Supporting Information.
