# Supplementary material for: Understanding changes in the locations of drinking occasions in Great Britain: An age‐period‐cohort analysis of repeat cross‐sectional market research data, 2001–2019
Source: Drug Alcohol Rev. 2022 Oct 12;42(1):105–18. doi: 10.1111/dar.13562 (PMC10092301; doi:10.1111/dar.13562)
Supplement: Supplementary file 1 — Appendix S1. Supporting Information. [file DAR-42-105-s001.docx]

## **Appendix 1. Additional details on methods**

*Details on ‘raking’ weighting technique:*

- First, the weighting process applies a weight to individuals such that the weighted distribution of social grade matches the distribution in the UK Census.
- Subsequently, an algorithm readjusts the weighted distribution based on geographic region, age and sex. The process is iterated repeatedly until the marginal distribution of all target variables has been matched with the UK Census data.

*Explanation of omission of 2008 from period measure in negative binomial regression models:*

- Within our 2001-2019 dataset, including both a period measure (categorised into 1-year intervals based on calendar year) and a binary control for the change in data collection (coded 0 = 2001-2008 on-street data collection, 1 = 2009-2019 online data collection) leads to perfect collinearity and what is known as the ‘dummy variable trap’.
- This is because our reference category is 2009, which allows us to estimate a value for the constant based on the observations for that year as all the other dummies take a value of 0. However, when we then introduce the binary control for change in data collection, which takes a value of 1 in 2009, we can no longer estimate the constant based on that year’s data, only the composite effect of the constant and the effect of the new dummy.
- Therefore, to estimate the constant we need to drop a dummy from the period 2001-2008 so that we can estimate the constant based on that year’s data. Therefore, 2008 is omitted as a category from the period measure (which year is dropped does not matter for the model, but does effect the coefficients as the constant is based on that year’s data).

## **Appendix 2. Detailed model results**

Table A2.1. Coefficient, standard error (SE) and *p*-value from negative binomial regression models for the age, period and cohort effects for men and women’s share (%) of weekly drinking occasions taking place in on-trade vs. off-trade locations, 2001-2019.

| **Location** |  | **Male** | | | **Female** | | |
| --- | --- | --- | --- | --- | --- | --- | --- |
|  |  | **Coefficient (B)** | **SE** | ***p*-value** | **Coefficient (B)** | **SE** | ***p*-value** |
| **On-trade** | **Age effects** | | | | | | |
|  | 18-24 | 0.167 | 0.015 | <0.001 | 0.365 | 0.024 | <0.001 |
|  | 25-34 | 0.083 | 0.011 | <0.001 | 0.138 | 0.017 | <0.001 |
|  | 35-39 | Reference category | | | | | |
|  | 50-64 | 0.018 | 0.014 | 0.187 | -0.011 | 0.022 | 0.626 |
|  | 65+ | 0.030 | 0.022 | 0.175 | -0.052 | 0.040 | 0.192 |
|  | **Period effects** | | | | | | |
|  | 2001 | 0.183 | 0.021 | <0.001 | 0.144 | 0.033 | <0.001 |
|  | 2002 | 0.135 | 0.022 | <0.001 | 0.099 | 0.034 | 0.004 |
|  | 2003 | 0.193 | 0.022 | <0.001 | 0.052 | 0.034 | 0.128 |
|  | 2004 | 0.149 | 0.022 | <0.001 | 0.043 | 0.034 | 0.212 |
|  | 2005 | 0.103 | 0.022 | <0.001 | 0.070 | 0.033 | 0.036 |
|  | 2006 | 0.091 | 0.021 | <0.001 | 0.067 | 0.033 | 0.043 |
|  | 2007 | 0.026 | 0.022 | 0.236 | -0.007 | 0.034 | 0.843 |
|  | 2008 | Omitted to remove perfect collinearity with control for change in data collection | | | | | |
|  | 2009 | Reference category | | | | | |
|  | 2010 | -0.009 | 0.017 | 0.575 | -0.103 | 0.031 | 0.001 |
|  | 2011 | -0.004 | 0.017 | 0.824 | -0.042 | 0.029 | 0.150 |
|  | 2012 | 0.009 | 0.017 | 0.577 | -0.048 | 0.029 | 0.104 |
|  | 2013 | -0.016 | 0.016 | 0.335 | -0.034 | 0.029 | 0.251 |
|  | 2014 | -0.027 | 0.017 | 0.104 | -0.022 | 0.028 | 0.447 |
|  | 2015 | -0.038 | 0.017 | 0.022 | 0.011 | 0.029 | 0.697 |
|  | 2016 | -0.039 | 0.017 | 0.020 | 0.006 | 0.030 | 0.854 |
|  | 2017 | -0.052 | 0.018 | 0.003 | -0.025 | 0.033 | 0.450 |
|  | 2018 | -0.088 | 0.017 | 0.000 | -0.048 | 0.033 | 0.143 |
|  | 2019 | -0.100 | 0.018 | 0.000 | -0.093 | 0.032 | 0.003 |
|  | **Cohort effects** | | | | | | |
|  | 1930s | -0.122 | 0.028 | <0.001 | -0.118 | 0.052 | 0.023 |
|  | 1940s | -0.026 | 0.019 | 0.168 | -0.040 | 0.031 | 0.199 |
|  | 1950s | 0.027 | 0.013 | 0.046 | 0.013 | 0.021 | 0.559 |
|  | 1960s | Reference category | | | | | |
|  | 1970s | 0.018 | 0.011 | 0.081 | 0.044 | 0.016 | 0.007 |
|  | 1980s | 0.111 | 0.015 | <0.001 | 0.187 | 0.024 | <0.001 |
|  | 1990s | 0.157 | 0.020 | <0.001 | 0.208 | 0.032 | <0.001 |
|  | **Control for change from on-street (2001-2008) to online (2009-2019) data collection** | | | | | | |
|  | On-street | Reference category | | | | | |
|  | Online | -0.204 | 0.020 | <0.001 | -0.181 | 0.032 | <0.001 |
| **Off-trade** | **Age effects** | | | | | | |
|  | 18-24 | -0.147 | 0.009 | <0.001 | -0.217 | 0.009 | <0.001 |
|  | 25-34 | -0.047 | 0.006 | <0.001 | -0.049 | 0.006 | <0.001 |
|  | 35-39 | Reference category | | | | | |
|  | 50-64 | -0.018 | 0.007 | 0.010 | -0.011 | 0.007 | 0.099 |
|  | 65+ | -0.031 | 0.011 | 0.007 | -0.018 | 0.012 | 0.123 |
|  | **Period effects** | | | | | | |
|  | 2001 | -0.134 | 0.014 | <0.001 | -0.073 | 0.013 | <0.001 |
|  | 2002 | -0.097 | 0.014 | <0.001 | -0.052 | 0.013 | <0.001 |
|  | 2003 | -0.139 | 0.015 | <0.001 | -0.035 | 0.013 | 0.008 |
|  | 2004 | -0.107 | 0.015 | <0.001 | -0.029 | 0.013 | 0.024 |
|  | 2005 | -0.071 | 0.014 | <0.001 | -0.037 | 0.013 | 0.004 |
|  | 2006 | -0.062 | 0.014 | <0.001 | -0.033 | 0.013 | 0.010 |
|  | 2007 | -0.019 | 0.014 | 0.173 | -0.003 | 0.013 | 0.823 |
|  | 2008 | Omitted to remove perfect collinearity with control for change in data collection | | | | | |
|  | 2009 | Reference category | | | | | |
|  | 2010 | 0.004 | 0.007 | 0.615 | 0.029 | 0.008 | <0.001 |
|  | 2011 | 0.000 | 0.008 | 0.973 | 0.011 | 0.008 | 0.158 |
|  | 2012 | -0.006 | 0.007 | 0.451 | 0.016 | 0.008 | 0.046 |
|  | 2013 | 0.005 | 0.007 | 0.486 | 0.013 | 0.008 | 0.113 |
|  | 2014 | 0.011 | 0.007 | 0.140 | 0.008 | 0.008 | 0.328 |
|  | 2015 | 0.015 | 0.007 | 0.043 | -0.001 | 0.008 | 0.858 |
|  | 2016 | 0.015 | 0.007 | 0.047 | -0.001 | 0.008 | 0.906 |
|  | 2017 | 0.021 | 0.008 | 0.008 | 0.008 | 0.009 | 0.376 |
|  | 2018 | 0.035 | 0.008 | <0.001 | 0.017 | 0.009 | 0.064 |
|  | 2019 | 0.040 | 0.008 | <0.001 | 0.028 | 0.009 | 0.001 |
|  | **Cohort effects** | | | | | | |
|  | 1930s | 0.071 | 0.013 | <0.001 | 0.078 | 0.014 | <0.001 |
|  | 1940s | 0.019 | 0.010 | 0.053 | 0.032 | 0.010 | 0.001 |
|  | 1950s | -0.006 | 0.007 | 0.379 | 0.005 | 0.007 | 0.437 |
|  | 1960s | Reference category | | | | | |
|  | 1970s | -0.017 | 0.005 | 0.001 | -0.024 | 0.005 | <0.001 |
|  | 1980s | -0.058 | 0.008 | <0.001 | -0.083 | 0.008 | <0.001 |
|  | 1990s | -0.035 | 0.011 | 0.001 | -0.043 | 0.011 | <0.001 |
|  | **Control for change from on-street (2001-2008) to online (2009-2019) data collection** | | | | | | |
|  | On-street | Reference category | | | | | |
|  | Online | 0.109 | 0.011 | <0.001 | 0.071 | 0.011 | <0.001 |

Table A2.2. Coefficient, standard error (SE) and *p*-value from negative binomial regression models for the age, period and cohort effects for men and women’s share (%) of weekly drinking occasions taking place in specific on-trade and off-trade locations, 2001-2019.

| **Location** |  | **Male** | | | **Female** | | |
| --- | --- | --- | --- | --- | --- | --- | --- |
|  |  | **Coefficient (B)** | **SE** | **p-value** | **Coefficient (B)** | **SE** | ***p*-value** |
| **Traditional/ community pub** | **Age effects** | | | | | | |
|  | 18-24 | 0.079 | 0.031 | 0.012 | 0.271 | 0.051 | <0.001 |
|  | 25-34 | 0.045 | 0.022 | 0.037 | 0.134 | 0.036 | <0.001 |
|  | 35-39 | Reference category | | | | | |
|  | 50-64 | 0.081 | 0.024 | 0.001 | -0.046 | 0.044 | 0.291 |
|  | 65+ | -0.007 | 0.040 | 0.868 | -0.268 | 0.083 | 0.001 |
|  | **Period effects** | | | | | | |
|  | 2001 | 0.182 | 0.037 | <0.001 | 0.301 | 0.069 | <0.001 |
|  | 2002 | 0.154 | 0.037 | <0.001 | 0.198 | 0.071 | 0.005 |
|  | 2003 | 0.220 | 0.038 | <0.001 | 0.226 | 0.070 | 0.001 |
|  | 2004 | 0.225 | 0.038 | <0.001 | 0.187 | 0.070 | 0.007 |
|  | 2005 | 0.110 | 0.038 | 0.004 | 0.065 | 0.067 | 0.327 |
|  | 2006 | 0.030 | 0.038 | 0.430 | 0.067 | 0.068 | 0.324 |
|  | 2007 | 0.049 | 0.039 | 0.205 | -0.026 | 0.071 | 0.718 |
|  | 2008 | Omitted to remove perfect collinearity with control for change in data collection | | | | | |
|  | 2009 | Reference category | | | | | |
|  | 2010 | -0.005 | 0.031 | 0.865 | -0.050 | 0.067 | 0.456 |
|  | 2011 | -0.043 | 0.033 | 0.190 | -0.051 | 0.065 | 0.427 |
|  | 2012 | 0.017 | 0.032 | 0.591 | -0.130 | 0.064 | 0.043 |
|  | 2013 | -0.056 | 0.032 | 0.078 | 0.022 | 0.067 | 0.747 |
|  | 2014 | -0.044 | 0.032 | 0.167 | 0.009 | 0.063 | 0.887 |
|  | 2015 | -0.101 | 0.032 | 0.002 | -0.039 | 0.066 | 0.549 |
|  | 2016 | -0.086 | 0.032 | 0.008 | 0.010 | 0.070 | 0.890 |
|  | 2017 | -0.073 | 0.035 | 0.036 | -0.039 | 0.073 | 0.590 |
|  | 2018 | -0.025 | 0.033 | 0.454 | 0.064 | 0.072 | 0.373 |
|  | 2019 | -0.047 | 0.034 | 0.173 | 0.043 | 0.068 | 0.529 |
|  | **Cohort effects** | | | | | | |
|  | 1930s | -0.430 | 0.052 | <0.001 | -0.243 | 0.116 | 0.036 |
|  | 1940s | -0.118 | 0.032 | <0.001 | -0.113 | 0.064 | 0.075 |
|  | 1950s | -0.013 | 0.023 | 0.533 | 0.067 | 0.043 | 0.116 |
|  | 1960s | Reference category | | | | | |
|  | 1970s | -0.135 | 0.019 | <0.001 | -0.057 | 0.033 | 0.085 |
|  | 1980s | -0.218 | 0.029 | <0.001 | -0.001 | 0.050 | 0.980 |
|  | 1990s | -0.274 | 0.041 | <0.001 | -0.114 | 0.070 | 0.102 |
|  | **Control for change from on-street (2001-2008) to online (2009-2019) data collection** | | | | | | |
|  | On-street | Reference category | | | | | |
|  | Online | -0.437 | 0.036 | <0.001 | -0.324 | 0.071 | <0.001 |
| **Modern pub/bar/**  **café bar** | **Age effects** | | | | | | |
|  | 18-24 | 0.213 | 0.048 | <0.001 | 0.517 | 0.089 | <0.001 |
|  | 25-34 | 0.160 | 0.031 | <0.001 | 0.252 | 0.055 | <0.001 |
|  | 35-39 | Reference category | | | | | |
|  | 50-64 | -0.098 | 0.048 | 0.041 | -0.172 | 0.078 | 0.028 |
|  | 65+ | -0.298 | 0.091 | 0.001 | -0.617 | 0.170 | <0.001 |
|  | **Period effects** | | | | | | |
|  | 2001 | 0.002 | 0.079 | 0.983 | -0.252 | 0.106 | 0.016 |
|  | 2002 | 0.074 | 0.082 | 0.366 | 0.045 | 0.118 | 0.703 |
|  | 2003 | 0.185 | 0.084 | 0.028 | -0.244 | 0.111 | 0.028 |
|  | 2004 | 0.133 | 0.083 | 0.111 | -0.034 | 0.119 | 0.776 |
|  | 2005 | 0.022 | 0.081 | 0.785 | -0.104 | 0.118 | 0.378 |
|  | 2006 | 0.206 | 0.080 | 0.010 | 0.001 | 0.118 | 0.996 |
|  | 2007 | -0.019 | 0.082 | 0.819 | -0.261 | 0.122 | 0.033 |
|  | 2008 | Omitted to remove perfect collinearity with control for change in data collection | | | | | |
|  | 2009 | Reference category | | | | | |
|  | 2010 | 0.055 | 0.061 | 0.362 | -0.187 | 0.148 | 0.206 |
|  | 2011 | 0.006 | 0.063 | 0.925 | -0.135 | 0.157 | 0.390 |
|  | 2012 | 0.076 | 0.060 | 0.207 | -0.186 | 0.143 | 0.194 |
|  | 2013 | 0.143 | 0.060 | 0.018 | -0.140 | 0.152 | 0.356 |
|  | 2014 | 0.098 | 0.058 | 0.092 | -0.216 | 0.138 | 0.117 |
|  | 2015 | 0.170 | 0.061 | 0.005 | -0.228 | 0.149 | 0.125 |
|  | 2016 | 0.042 | 0.058 | 0.470 | -0.255 | 0.140 | 0.069 |
|  | 2017 | 0.143 | 0.064 | 0.026 | -0.122 | 0.154 | 0.427 |
|  | 2018 | 0.023 | 0.058 | 0.693 | -0.297 | 0.184 | 0.107 |
|  | 2019 | 0.061 | 0.063 | 0.331 | -0.298 | 0.137 | 0.030 |
|  | **Cohort effects** | | | | | | |
|  | 1930s | -0.889 | 0.128 | <0.001 | -0.429 | -0.429 | 0.093 |
|  | 1940s | -0.670 | 0.073 | <0.001 | -0.518 | -0.518 | <0.001 |
|  | 1950s | -0.324 | 0.047 | <0.001 | -0.332 | -0.332 | <0.001 |
|  | 1960s | Reference category | | | | | |
|  | 1970s | 0.321 | 0.032 | <0.001 | 0.355 | 0.255 | <0.001 |
|  | 1980s | 0.565 | 0.047 | <0.001 | 0.656 | 0.656 | <0.001 |
|  | 1990s | 0.531 | 0.067 | <0.001 | 0.631 | 0.631 | <0.001 |
|  | **Control for change from on-street (2001-2008) to online (2009-2019) data collection** | | | | | | |
|  | On-street | Reference category | | | | | |
|  | Online | -0.780 | 0.071 | <0.001 | -0.737 | 0.142 | <0.001 |
| **Nightclub/**  **late-night venue** | **Age effects** | | | | | | |
|  | 18-24 | 0.582 | 0.125 | <0.001 | 0.856 | 0.166 | <0.001 |
|  | 25-34 | 0.138 | 0.080 | 0.083 | 0.230 | 0.111 | 0.037 |
|  | 35-39 | Reference category | | | | | |
|  | 50-64 | -0.310 | 0.145 | 0.033 | -0.121 | 0.192 | 0.528 |
|  | 65+ | -0.732 | 0.355 | 0.039 | -1.179 | 0.536 | 0.028 |
|  | **Period effects** | | | | | | |
|  | 2001 | 0.538 | 0.407 | 0.187 | 0.768 | 0.210 | <0.001 |
|  | 2002 | 0.488 | 0.376 | 0.195 | 1.629 | 0.551 | 0.003 |
|  | 2003 | 0.607 | 0.443 | 0.171 | 0.833 | 0.277 | 0.003 |
|  | 2004 | -0.492 | 0.321 | 0.125 | 0.838 | 0.266 | 0.002 |
|  | 2005 | -0.532 | 0.315 | 0.091 | 0.804 | 0.566 | 0.156 |
|  | 2006 | 0.255 | 0.498 | 0.609 | 0.669 | 0.286 | 0.019 |
|  | 2007 | -0.368 | 0.369 | 0.319 | 0.164 | 0.233 | 0.482 |
|  | 2008 | Omitted to remove perfect collinearity with control for change in data collection | | | | | |
|  | 2009 | Reference category | | | | | |
|  | 2010 | -0.207 | 0.183 | 0.258 | -0.044 | 0.178 | 0.805 |
|  | 2011 | 0.016 | 0.265 | 0.953 | 1.094 | 0.440 | 0.013 |
|  | 2012 | -0.354 | 0.182 | 0.052 | -0.320 | 0.181 | 0.077 |
|  | 2013 | -0.225 | 0.185 | 0.225 | 0.825 | 0.632 | 0.191 |
|  | 2014 | -0.155 | 0.223 | 0.606 | -0.314 | 0.175 | 0.073 |
|  | 2015 | -0.471 | 0.182 | 0.010 | -0.531 | 0.191 | 0.005 |
|  | 2016 | -0.286 | 0.254 | 0.261 | -0.277 | 0.239 | 0.247 |
|  | 2017 | -0.479 | 0.233 | 0.040 | -0.359 | 0.214 | 0.094 |
|  | 2018 | -0.868 | 0.193 | 0.000 | -0.690 | 0.208 | 0.001 |
|  | 2019 | -0.456 | 0.206 | 0.027 | -0.427 | 0.207 | 0.039 |
|  | **Cohort effects** | | | | | | |
|  | 1930s | -2.266 | 0.448 | <0.001 | -2.063 | 0.723 | 0.004 |
|  | 1940s | -2.026 | 0.301 | <0.001 | -1.582 | 0.385 | <0.001 |
|  | 1950s | -0.613 | 0.146 | <0.001 | -0.596 | 0.204 | 0.003 |
|  | 1960s | Reference category | | | | | |
|  | 1970s | 0.872 | 0.083 | <0.001 | 0.898 | 0.119 | <0.001 |
|  | 1980s | 1.566 | 0.125 | <0.001 | 1.687 | 0.173 | <0.001 |
|  | 1990s | 1.958 | 0.178 | <0.001 | 2.214 | 0.242 | <0.001 |
|  | **Control for change from on-street (2001-2008) to online (2009-2019) data collection** | | | | | | |
|  | On-street | Reference category | | | | | |
|  | Online | 0.157 | 0.334 | 0.638 | 0.175 | 0.197 | 0.373 |
| **Restaurant/ pub restaurant** | **Age effects** | | | | | | |
|  | 18-24 | 0.051 | 0.047 | 0.279 | 0.066 | 0.050 | 0.190 |
|  | 25-34 | 0.170 | 0.033 | <0.001 | 0.143 | 0.037 | <0.001 |
|  | 35-39 | Reference category | | | | | |
|  | 50-64 | -0.099 | 0.041 | 0.015 | -0.013 | 0.043 | 0.757 |
|  | 65+ | -0.055 | 0.063 | 0.383 | -0.002 | 0.072 | 0.975 |
|  | **Period effects** | | | | | | |
|  | 2001 | 0.092 | 0.085 | 0.278 | 0.065 | 0.082 | 0.429 |
|  | 2002 | 0.066 | 0.086 | 0.445 | -0.023 | 0.084 | 0.786 |
|  | 2003 | 0.172 | 0.086 | 0.045 | -0.088 | 0.084 | 0.293 |
|  | 2004 | 0.043 | 0.086 | 0.615 | -0.094 | 0.084 | 0.263 |
|  | 2005 | 0.033 | 0.085 | 0.699 | 0.077 | 0.081 | 0.342 |
|  | 2006 | 0.197 | 0.083 | 0.017 | 0.141 | 0.078 | 0.071 |
|  | 2007 | 0.051 | 0.085 | 0.546 | 0.024 | 0.080 | 0.765 |
|  | 2008 | Omitted to remove perfect collinearity with control for change in data collection | | | | | |
|  | 2009 | Reference category | | | | | |
|  | 2010 | 0.015 | 0.039 | 0.709 | -0.133 | 0.051 | 0.010 |
|  | 2011 | 0.028 | 0.039 | 0.474 | -0.019 | 0.047 | 0.691 |
|  | 2012 | 0.048 | 0.039 | 0.215 | 0.004 | 0.047 | 0.939 |
|  | 2013 | 0.080 | 0.038 | 0.035 | -0.077 | 0.047 | 0.099 |
|  | 2014 | 0.082 | 0.038 | 0.032 | -0.053 | 0.046 | 0.248 |
|  | 2015 | 0.122 | 0.038 | 0.001 | 0.068 | 0.046 | 0.142 |
|  | 2016 | 0.123 | 0.038 | 0.001 | 0.030 | 0.048 | 0.526 |
|  | 2017 | 0.079 | 0.042 | 0.058 | 0.015 | 0.053 | 0.771 |
|  | 2018 | 0.004 | 0.041 | 0.923 | -0.037 | 0.054 | 0.492 |
|  | 2019 | -0.063 | 0.043 | 0.147 | -0.131 | 0.052 | 0.012 |
|  | **Cohort effects** | | | | | | |
|  | 1930s | 0.207 | 0.074 | 0.005 | 0.067 | 0.093 | 0.474 |
|  | 1940s | 0.285 | 0.054 | <0.001 | 0.224 | 0.059 | <0.001 |
|  | 1950s | 0.206 | 0.038 | <0.001 | 0.159 | 0.041 | <0.001 |
|  | 1960s | Reference category | | | | | |
|  | 1970s | -0.028 | 0.031 | 0.359 | -0.090 | 0.033 | 0.006 |
|  | 1980s | -0.034 | 0.045 | 0.448 | -0.052 | 0.049 | 0.288 |
|  | 1990s | 0.173 | 0.060 | 0.004 | 0.199 | 0.065 | 0.002 |
|  | **Control for change from on-street (2001-2008) to online (2009-2019) data collection** | | | | | | |
|  | On-street | Reference category | | | | | |
|  | Online | 0.703 | 0.068 | <0.001 | 0.469 | 0.068 | <0.001 |
| **Social/**  **working men’s club** | **Age effects** | | | | | | |
|  | 18-24 | -0.029 | 0.124 | 0.818 | -0.208 | 0.125 | 0.185 |
|  | 25-34 | -0.004 | 0.078 | 0.961 | -0.147 | 0.157 | 0.173 |
|  | 35-39 | Reference category | | | | | |
|  | 50-64 | 0.387 | 0.066 | <0.001 | 0.130 | 0.100 | 0.196 |
|  | 65+ | 0.699 | 0.098 | <0.001 | 0.260 | 0.174 | 0.135 |
|  | **Period effects** | | | | | | |
|  | 2001 | 0.337 | 0.089 | <0.001 | 0.168 | 0.123 | 0.172 |
|  | 2002 | 0.163 | 0.093 | 0.080 | 0.146 | 0.123 | 0.235 |
|  | 2003 | 0.195 | 0.094 | 0.038 | 0.044 | 0.128 | 0.729 |
|  | 2004 | 0.064 | 0.096 | 0.508 | -0.004 | 0.127 | 0.978 |
|  | 2005 | 0.285 | 0.093 | 0.002 | 0.184 | 0.120 | 0.126 |
|  | 2006 | 0.199 | 0.093 | 0.031 | 0.022 | 0.124 | 0.860 |
|  | 2007 | 0.114 | 0.096 | 0.237 | 0.194 | 0.119 | 0.105 |
|  | 2008 | Omitted to remove perfect collinearity with control for change in data collection | | | | | |
|  | 2009 | Reference category | | | | | |
|  | 2010 | -0.077 | 0.083 | 0.353 | 0.060 | 0.129 | 0.640 |
|  | 2011 | -0.039 | 0.080 | 0.624 | -0.020 | 0.129 | 0.879 |
|  | 2012 | -0.037 | 0.082 | 0.648 | -0.077 | 0.129 | 0.552 |
|  | 2013 | -0.272 | 0.089 | 0.002 | 0.004 | 0.132 | 0.977 |
|  | 2014 | -0.380 | 0.090 | <0.001 | 0.057 | 0.132 | 0.664 |
|  | 2015 | -0.439 | 0.090 | <0.001 | -0.023 | 0.136 | 0.864 |
|  | 2016 | -0.344 | 0.089 | <0.001 | -0.030 | 0.141 | 0.833 |
|  | 2017 | -0.584 | 0.101 | <0.001 | -0.120 | 0.153 | 0.434 |
|  | 2018 | -0.414 | 0.104 | <0.001 | 0.055 | 0.150 | 0.713 |
|  | 2019 | -0.513 | 0.015 | <0.001 | -0.105 | 0.161 | 0.514 |
|  | **Cohort effects** | | | | | | |
|  | 1930s | 0.306 | 0.109 | 0.005 | 0.372 | 0.200 | 0.063 |
|  | 1940s | 0.416 | 0.078 | <0.001 | 0.256 | 0.137 | 0.062 |
|  | 1950s | 0.273 | 0.060 | <0.001 | 0.077 | 0.096 | 0.420 |
|  | 1960s | Reference category | | | | | |
|  | 1970s | -0.588 | 0.065 | <0.001 | -0.366 | 0.094 | <0.001 |
|  | 1980s | -0.779 | 0.102 | <0.001 | -0.457 | 0.146 | 0.002 |
|  | 1990s | -1.204 | 0.163 | <0.001 | -0.674 | 0.201 | 0.001 |
|  | **Control for change from on-street (2001-2008) to online (2009-2019) data collection** | | | | | | |
|  | On-street | Reference category | | | | | |
|  | Online | -0.367 | 0.090 | <0.001 | -0.920 | 0.125 | <0.001 |
| **Golf/other sports club/venue** | **Age effects** | | | | | | |
|  | 18-24 | -0.059 | 0.142 | 0.678 | -0.468 | 0.222 | 0.035 |
|  | 25-34 | -0.083 | 0.098 | 0.400 | -0.227 | 0.157 | 0.149 |
|  | 35-39 | Reference category | | | | | |
|  | 50-64 | 0.065 | 0.098 | 0.508 | -0.238 | 0.199 | 0.230 |
|  | 65+ | 0.470 | 0.141 | 0.001 | -0.613 | 0.334 | 0.066 |
|  | **Period effects** | | | | | | |
|  | 2001 | -0.376 | 0.148 | 0.011 | -0.184 | 0.258 | 0.476 |
|  | 2002 | -0.343 | 0.151 | 0.024 | -0.385 | 0.267 | 0.149 |
|  | 2003 | -0.611 | 0.157 | <0.001 | -0.592 | 0.313 | 0.059 |
|  | 2004 | -0.288 | 0.155 | 0.062 | -0.522 | 0.282 | 0.064 |
|  | 2005 | -0.299 | 0.149 | 0.045 | -0.347 | 0.264 | 0.190 |
|  | 2006 | -0.169 | 0.146 | 0.246 | -0.146 | 0.258 | 0.572 |
|  | 2007 | -0.518 | 0.160 | 0.001 | -0.536 | 0.312 | 0.086 |
|  | 2008 | Omitted to remove perfect collinearity with control for change in data collection | | | | | |
|  | 2009 | Reference category | | | | | |
|  | 2010 | 0.015 | 0.093 | 0.870 | 0.064 | 0.208 | 0.758 |
|  | 2011 | 0.059 | 0.097 | 0.546 | 0.159 | 0.183 | 0.385 |
|  | 2012 | -0.252 | 0.097 | 0.009 | 0.048 | 0.179 | 0.786 |
|  | 2013 | -0.172 | 0.103 | 0.095 | 0.221 | 0.191 | 0.247 |
|  | 2014 | -0.443 | 0.102 | <0.001 | -0.129 | 0.222 | 0.562 |
|  | 2015 | -0.311 | 0.105 | 0.003 | 0.026 | 0.197 | 0.893 |
|  | 2016 | -0.224 | 0.107 | 0.037 | -0.171 | 0.213 | 0.423 |
|  | 2017 | -0.567 | 0.115 | <0.001 | -0.566 | 0.233 | 0.015 |
|  | 2018 | -0.516 | 0.124 | <0.001 | -0.208 | 0.244 | 0.394 |
|  | 2019 | -0.451 | 0.120 | <0.001 | -0.057 | 0.236 | 0.808 |
|  | **Cohort effects** | | | | | | |
|  | 1930s | 0.102 | 0.160 | 0.522 | 0.850 | 0.381 | 0.026 |
|  | 1940s | 0.255 | 0.121 | 0.034 | 0.247 | 0.263 | 0.347 |
|  | 1950s | 0.274 | 0.092 | 0.003 | 0.097 | 0.198 | 0.626 |
|  | 1960s | Reference category | | | | | |
|  | 1970s | -0.186 | 0.081 | 0.022 | -0.053 | 0.138 | 0.702 |
|  | 1980s | -0.193 | 0.130 | 0.136 | 0.091 | 0.215 | 0.672 |
|  | 1990s | -0.348 | 0.179 | 0.051 | 0.171 | 0.291 | 0.556 |
|  | **Control for change from on-street (2001-2008) to online (2009-2019) data collection** | | | | | | |
|  | On-street | Reference category | | | | | |
|  | Online | -0.156 | 0.123 | 0.204 | -0.492 | 0.228 | 0.031 |
| **At home (social setting)** | **Age effects** | | | | | | |
|  | 18-24 | -0.025 | 0.029 | 0.390 | -0.086 | 0.030 | 0.004 |
|  | 25-34 | 0.080 | 0.020 | <0.001 | 0.031 | 0.021 | 0.145 |
|  | 35-39 | Reference category | | | | | |
|  | 50-64 | -0.213 | 0.027 | <0.001 | -0.006 | 0.027 | 0.818 |
|  | 65+ | -0.351 | 0.045 | <0.001 | -0.004 | 0.048 | 0.939 |
|  | **Period effects** | | | | | | |
|  | 2001 | 0.004 | 0.049 | 0.930 | -0.017 | 0.045 | 0.709 |
|  | 2002 | -0.090 | 0.051 | 0.078 | -0.039 | 0.046 | 0.398 |
|  | 2003 | -0.053 | 0.050 | 0.290 | -0.037 | 0.046 | 0.426 |
|  | 2004 | 0.034 | 0.050 | 0.489 | -0.070 | 0.046 | 0.130 |
|  | 2005 | 0.057 | 0.048 | 0.231 | -0.014 | 0.043 | 0.754 |
|  | 2006 | -0.116 | 0.049 | 0.018 | -0.060 | 0.044 | 0.168 |
|  | 2007 | -0.092 | 0.050 | 0.062 | -0.050 | 0.045 | 0.262 |
|  | 2008 | Omitted to remove perfect collinearity with control for change in data collection | | | | | |
|  | 2009 | Reference category | | | | | |
|  | 2010 | -0.021 | 0.028 | 0.470 | -0.060 | 0.032 | 0.061 |
|  | 2011 | -0.006 | 0.029 | 0.825 | -0.029 | 0.032 | 0.358 |
|  | 2012 | -0.015 | 0.029 | 0.591 | -0.092 | 0.032 | 0.004 |
|  | 2013 | -0.058 | 0.029 | 0.043 | -0.104 | 0.032 | 0.001 |
|  | 2014 | -0.075 | 0.029 | 0.010 | -0.130 | 0.032 | <0.001 |
|  | 2015 | -0.105 | 0.029 | <0.001 | -0.150 | 0.033 | <0.001 |
|  | 2016 | -0.092 | 0.029 | 0.002 | -0.166 | 0.034 | <0.001 |
|  | 2017 | -0.151 | 0.033 | <0.001 | -0.154 | 0.038 | <0.001 |
|  | 2018 | -0.096 | 0.030 | 0.001 | -0.135 | 0.037 | <0.001 |
|  | 2019 | -0.073 | 0.032 | 0.021 | -0.165 | 0.035 | <0.001 |
|  | **Cohort effects** | | | | | | |
|  | 1930s | 0.012 | 0.055 | 0.823 | -0.179 | 0.061 | 0.003 |
|  | 1940s | 0.005 | 0.038 | 0.903 | -0.035 | 0.038 | 0.362 |
|  | 1950s | -0.004 | 0.026 | 0.889 | -0.043 | 0.027 | 0.105 |
|  | 1960s | Reference category | | | | | |
|  | 1970s | 0.065 | 0.019 | 0.001 | 0.059 | 0.020 | 0.003 |
|  | 1980s | 0.251 | 0.028 | <0.001 | 0.128 | 0.030 | <0.001 |
|  | 1990s | 0.491 | 0.038 | <0.001 | 0.327 | 0.041 | <0.001 |
|  | **Control for change from on-street (2001-2008) to online (2009-2019) data collection** | | | | | | |
|  | On-street | Reference category | | | | | |
|  | Online | 0.172 | 0.040 | <0.001 | 0.056 | 0.039 | 0.149 |
| **At home**  **(non-social setting)** | **Age effects** | | | | | | |
|  | 18-24 | -0.246 | 0.015 | <0.001 | -0.030 | 0.016 | <0.001 |
|  | 25-34 | -0.121 | 0.010 | <0.001 | -0.104 | 0.011 | <0.001 |
|  | 35-39 | Reference category | | | | | |
|  | 50-64 | 0.031 | 0.010 | 0.004 | -0.019 | 0.012 | 0.116 |
|  | 65+ | 0.028 | 0.017 | 0.093 | -0.051 | 0.021 | 0.014 |
|  | **Period effects** | | | | | | |
|  | 2001 | -0.167 | 0.021 | <0.001 | -0.097 | 0.022 | <0.001 |
|  | 2002 | -0.096 | 0.020 | <0.001 | -0.051 | 0.022 | 0.019 |
|  | 2003 | -0.156 | 0.021 | <0.001 | -0.026 | 0.022 | 0.224 |
|  | 2004 | -0.150 | 0.021 | <0.001 | -0.022 | 0.022 | 0.307 |
|  | 2005 | -0.096 | 0.020 | <0.001 | -0.046 | 0.021 | 0.033 |
|  | 2006 | -0.063 | 0.020 | 0.001 | -0.021 | 0.021 | 0.313 |
|  | 2007 | 0.002 | 0.019 | 0.919 | 0.021 | 0.021 | 0.322 |
|  | 2008 | Omitted to Remove Perfect Collinearity with Control for Change in Data Collection | | | | | |
|  | 2009 | Reference category | | | | | |
|  | 2010 | 0.019 | 0.012 | 0.102 | 0.055 | 0.014 | <0.001 |
|  | 2011 | 0.009 | 0.012 | 0.448 | 0.020 | 0.015 | 0.168 |
|  | 2012 | 0.011 | 0.012 | 0.358 | 0.058 | 0.014 | <0.001 |
|  | 2013 | 0.029 | 0.011 | 0.011 | 0.067 | 0.014 | <0.001 |
|  | 2014 | 0.042 | 0.012 | <0.001 | 0.070 | 0.014 | <0.001 |
|  | 2015 | 0.057 | 0.012 | <0.001 | 0.066 | 0.015 | <0.001 |
|  | 2016 | 0.055 | 0.012 | <0.001 | 0.074 | 0.015 | <0.001 |
|  | 2017 | 0.073 | 0.013 | <0.001 | 0.074 | 0.016 | <0.001 |
|  | 2018 | 0.070 | 0.012 | <0.001 | 0.071 | 0.016 | <0.001 |
|  | 2019 | 0.061 | 0.013 | <0.001 | 0.089 | 0.016 | <0.001 |
|  | **Cohort effects** | | | | | | |
|  | 1930s | 0.114 | 0.019 | <0.001 | 0.198 | 0.025 | <0.001 |
|  | 1940s | 0.028 | 0.014 | 0.048 | 0.066 | 0.017 | <0.001 |
|  | 1950s | -0.009 | 0.010 | 0.378 | 0.020 | 0.011 | 0.085 |
|  | 1960s | Reference category | | | | | |
|  | 1970s | -0.025 | 0.008 | 0.002 | -0.047 | 0.009 | <0.001 |
|  | 1980s | -0.140 | 0.013 | <0.001 | -0.143 | 0.014 | <0.001 |
|  | 1990s | -0.220 | 0.019 | <0.001 | -0.165 | 0.020 | <0.001 |
|  | **Control for change from on-street (2001-2008) to online (2009-2019) data collection** | | | | | | |
|  | On-street | Reference category | | | | | |
|  | Online | 0.107 | 0.016 | <0.001 | 0.087 | 0.019 | <0.001 |

## **Appendix 3. Sensitivity analysis 1 – 2001-2008/2009-2019 modelled separately**

Figure B1. Age, period and cohort effects on percentage share of men and women’s individual drinking occasions per week taking place in on-trade vs. off-trade locations, 2001-2008 and 2009-2019. Blue: men; orange: women.

**
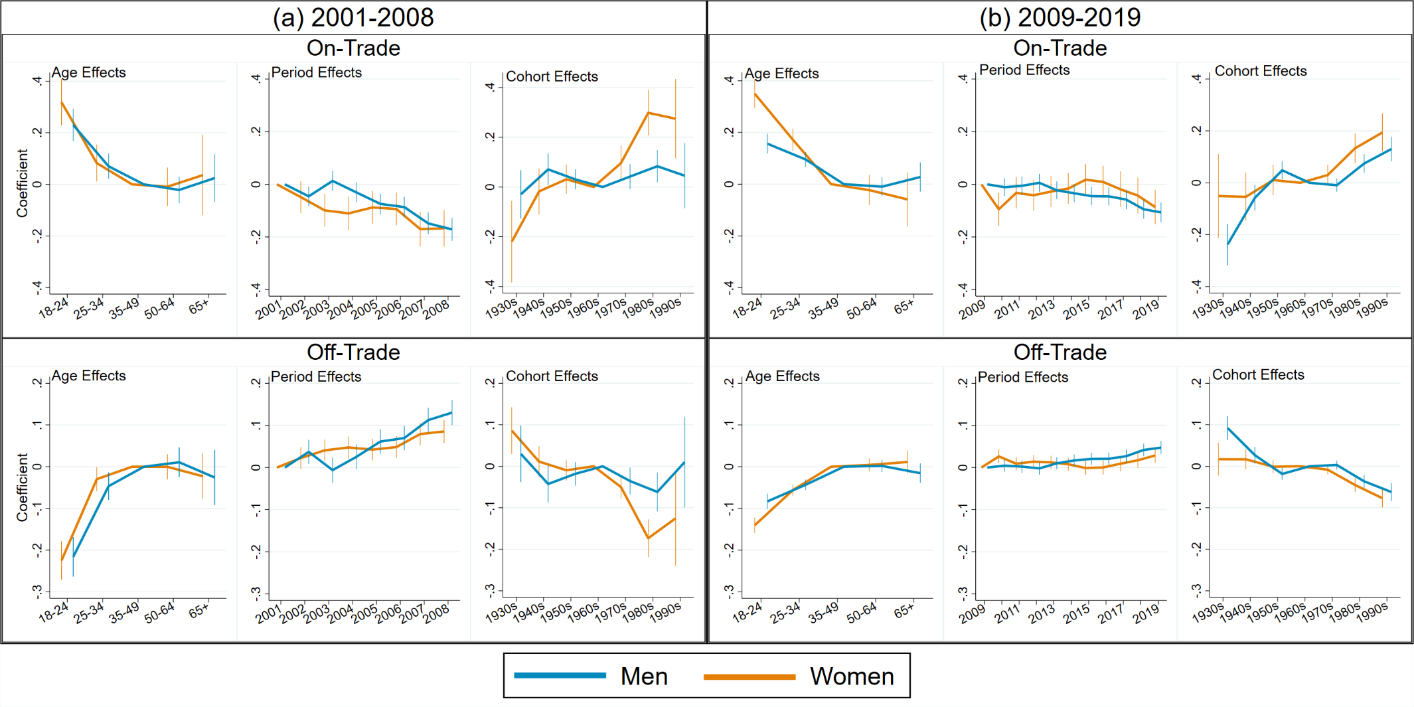
**

*Notes*: different locations have different y-axis ranges to best depict results. Vertical bars represent 95% confidence intervals at each data point. Reference categories: 35-49, 2001, 2009 and 1960s.

Figure B2. Age, period and cohort effects on percentage share of men and women’s individual drinking occasions per week taking place in specific on-trade and off-trade locations, 2001-2008 and 2009-2019. Blue: men; orange: women.

**
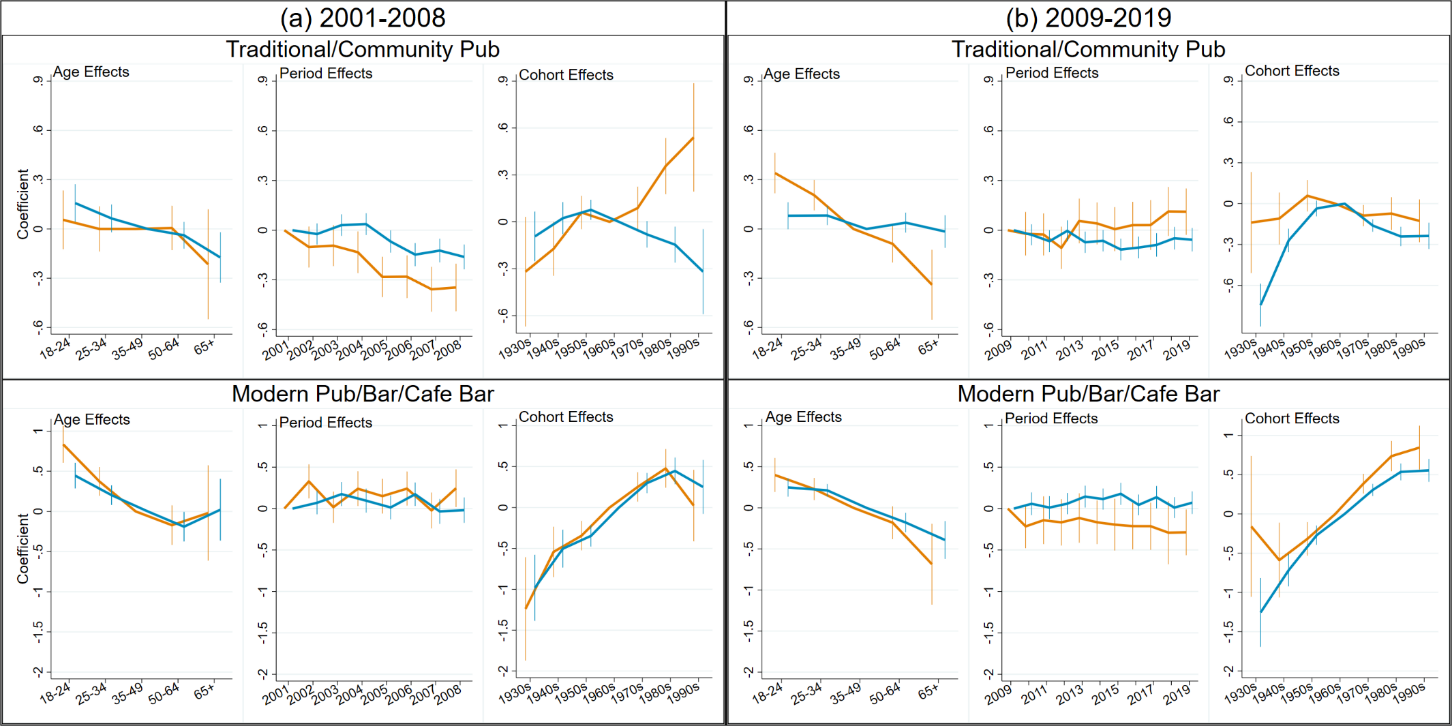
**

Figure B2 (continued)

*
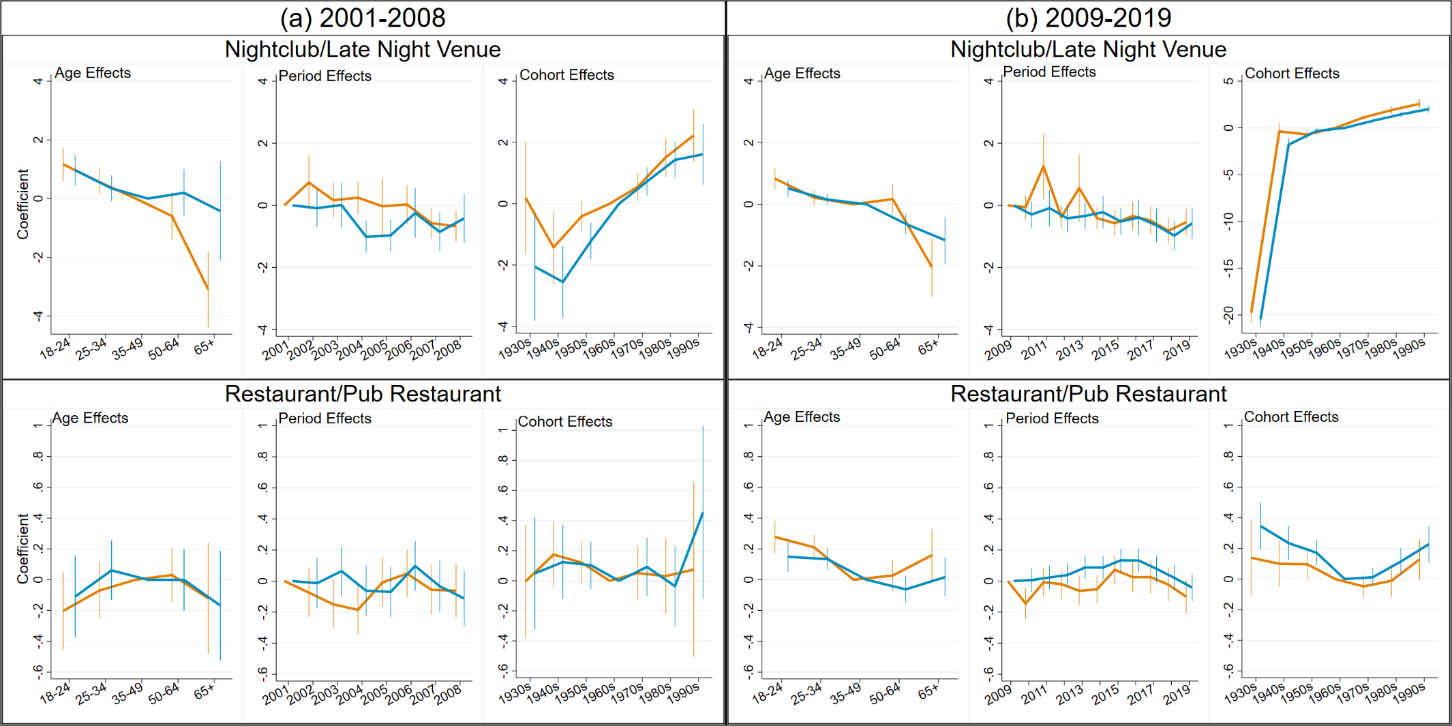
*

Figure B2 (continued)

*
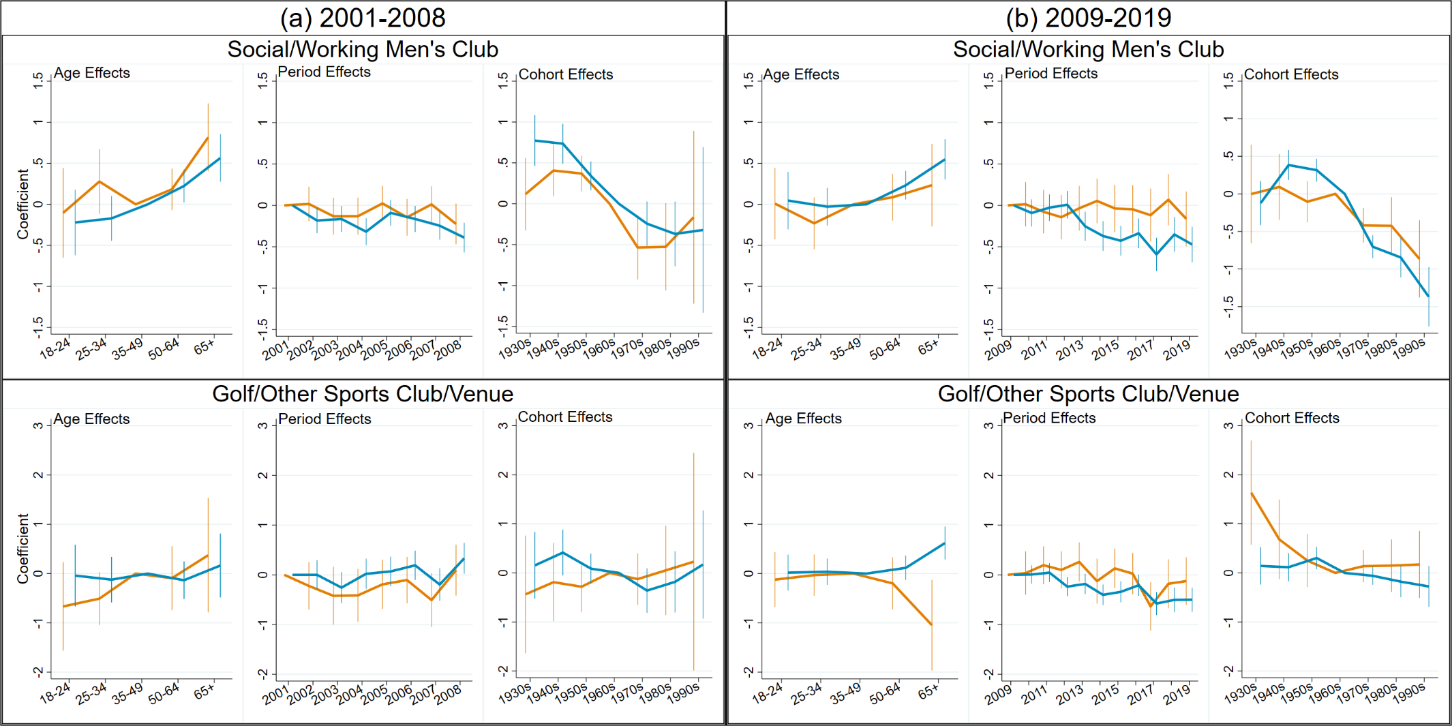
*

Figure B2 (continued)

*
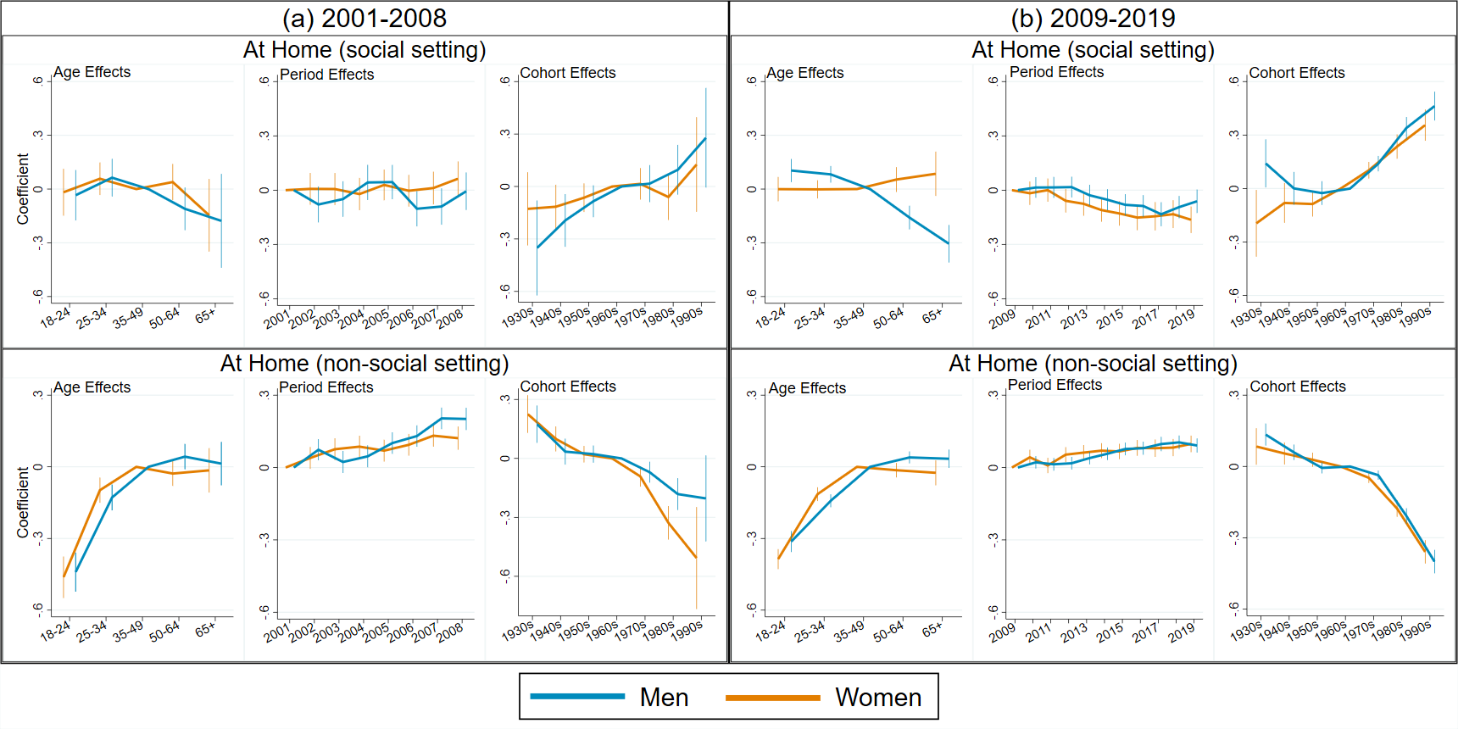
*

*Notes*: *Notes*: different locations have different y-axis ranges to best depict results. Vertical bars represent 95% confidence intervals at each data point. Reference categories: 35-49, 2001, 2009 and 1960s. The large drop off for nightclub/late-night venue occasions for the 1930s birth cohort reflects the fact that this type of occasion was extremely rare for this group in the sample

## **Appendix 4. Sensitivity analysis 2 –** **Age-period-cohort variables in models specified differently**

Figure C1. Age, period and cohort effects on percentage share of men and women’s individual drinking occasions per week taking place in on-trade vs. off-trade locations, 2001/2002 – 2018/2019. Blue: men; orange: women.

**
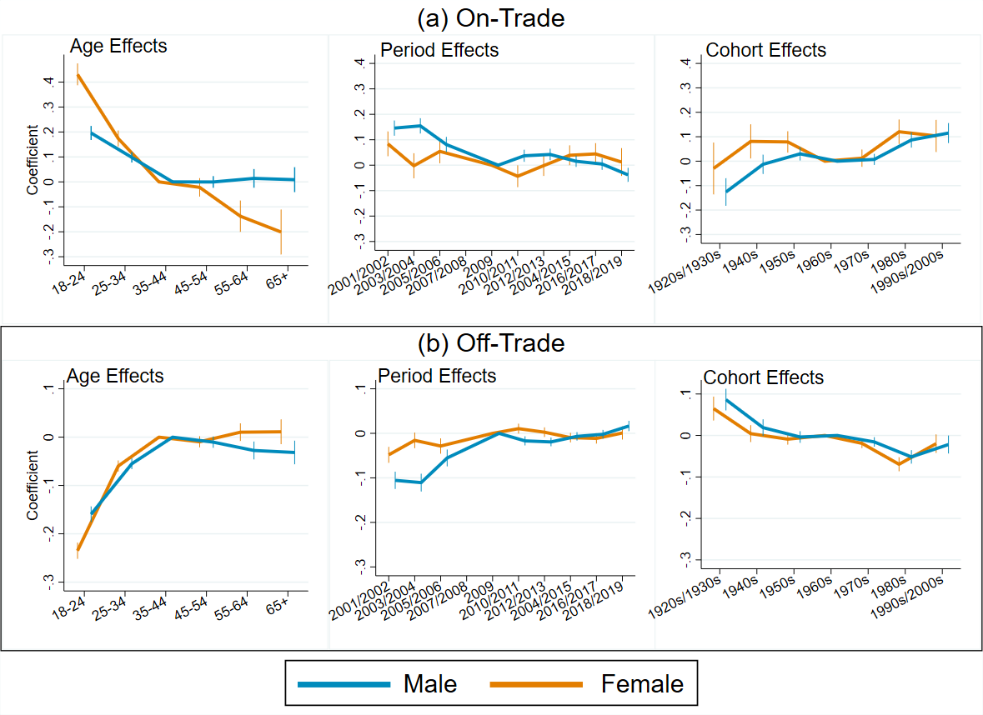
**

*Notes*: different locations have different y-axis ranges to best depict results. Vertical bars represent 95% confidence intervals at each data point. Reference categories: 35-44, 2009 and 1960s. 2007/2008 was omitted from the negative binomial regression model to remove perfect collinearity between period variable and the binary control for the change in data collection between 2008 and 2009.

Figure C2. Age, period and cohort effects on percentage share of men and women’s individual drinking occasions per week taking place in specific on-trade and off-trade locations, 2001/2002-2018/2019. Blue: men; orange: women.

**
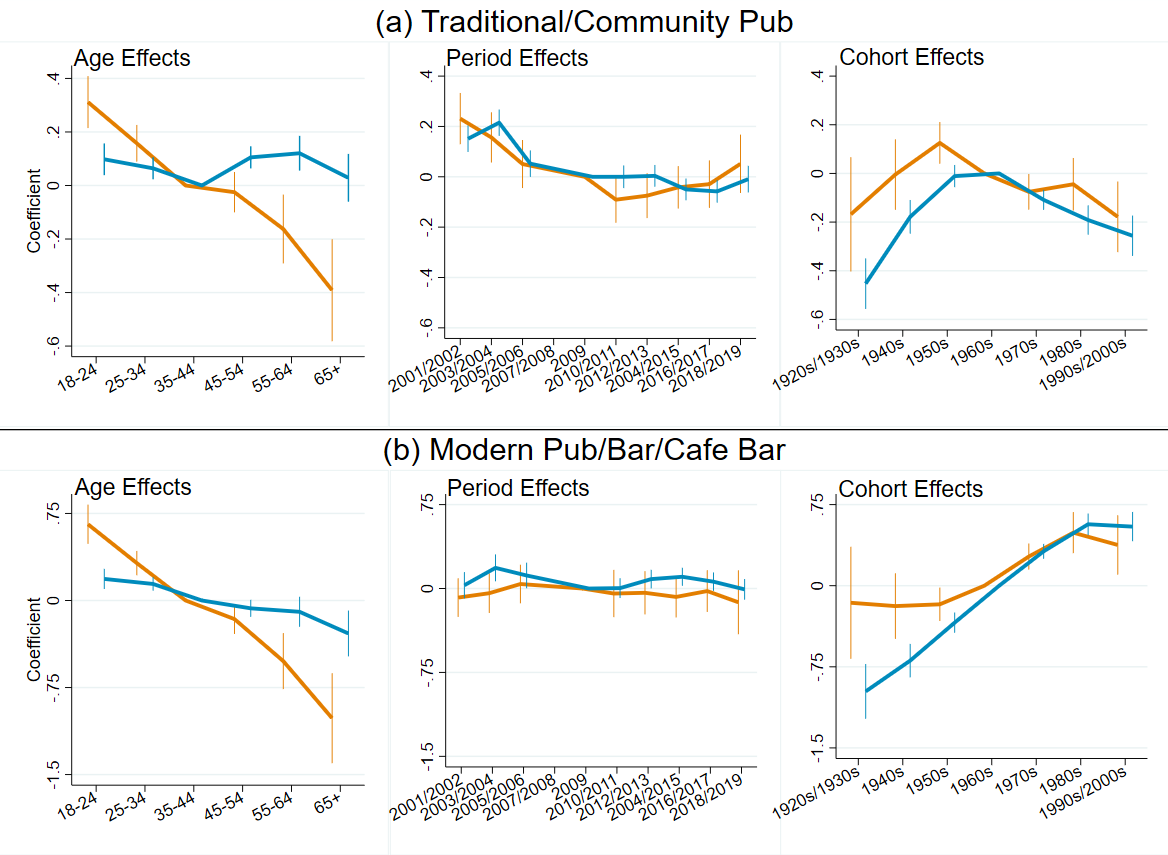
**

Figure C2 (continued)

*
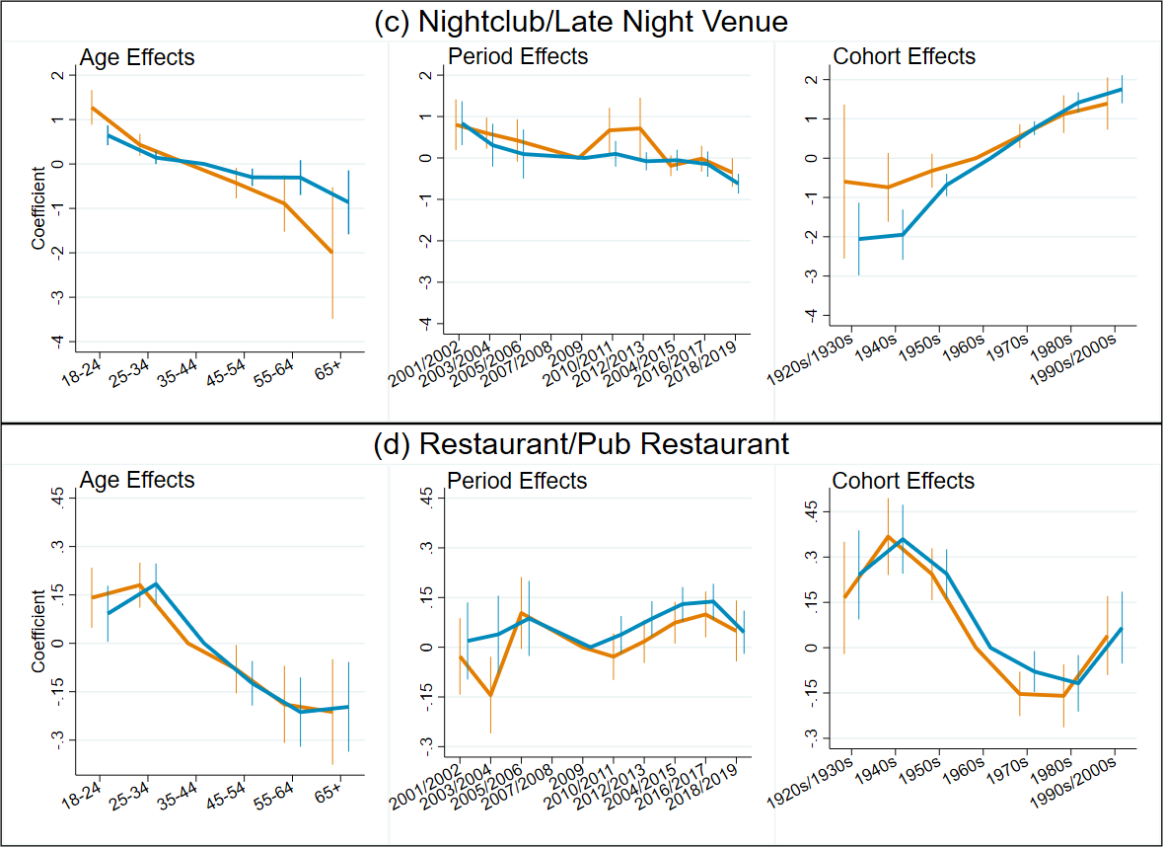
*

Figure C2 (continued)


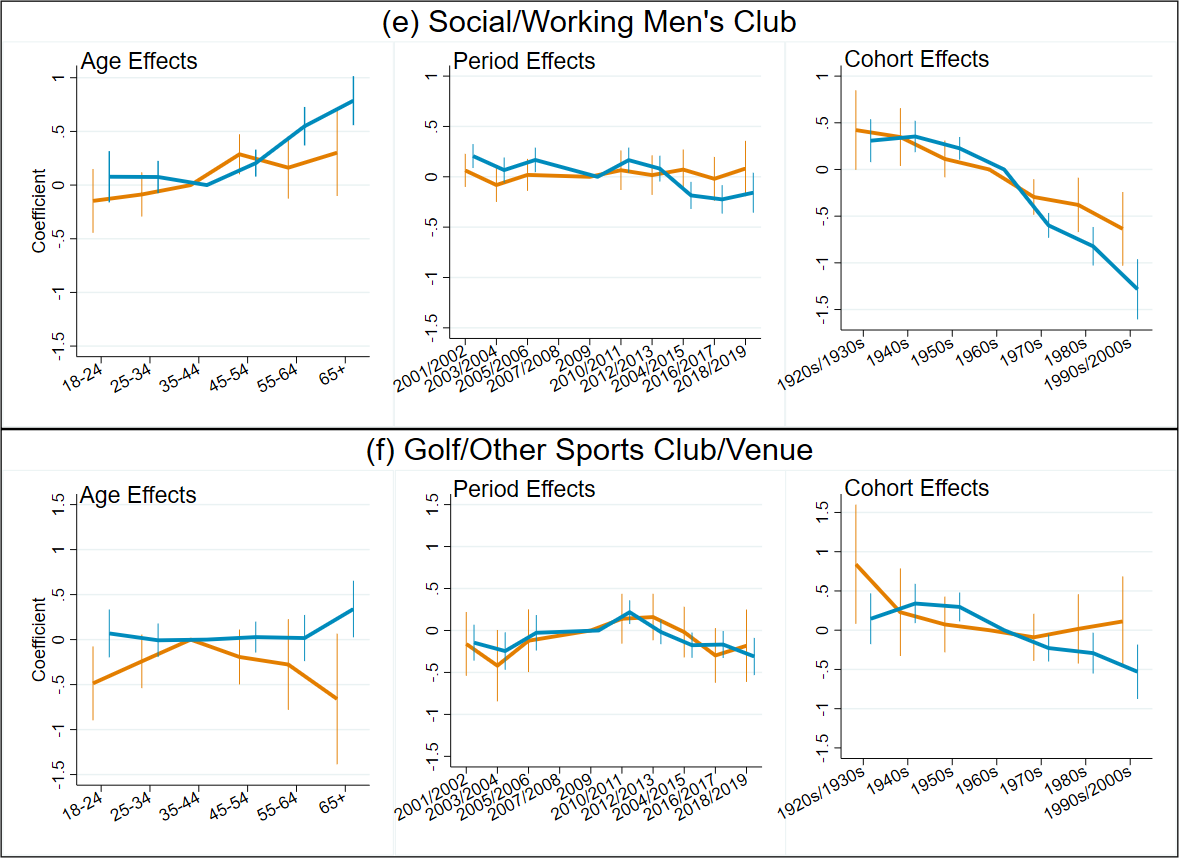


Figure C2 (continued)

*
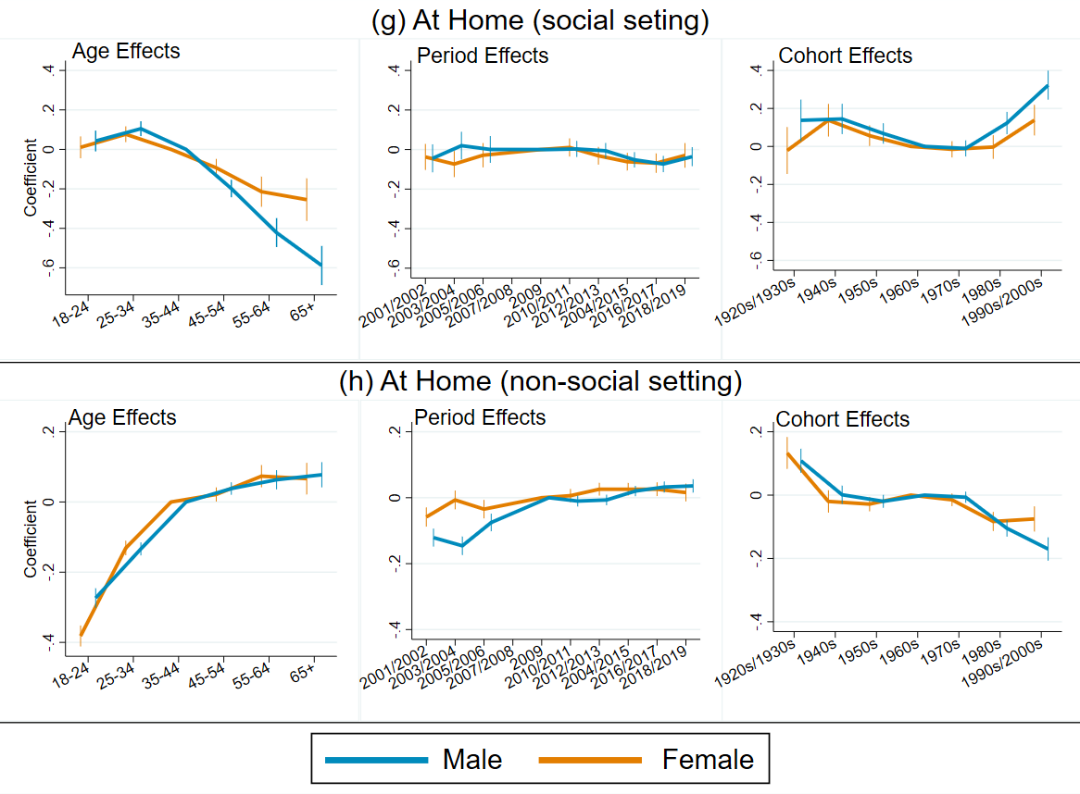
*

*Notes*: plots have varying y-axis ranges to best depict results. Vertical bars represent 95% confidence intervals at each data point. Reference categories: 35-44, 2009 and 1960s. 2007/2008 was omitted from the negative binomial regression model to remove perfect collinearity between period variable and the binary control for the change in data collection between 2008 and 2009.
